# Revealing the uncharacterised diversity of amphibian and reptile viruses

**DOI:** 10.1038/s43705-022-00180-x

**Published:** 2022-10-02

**Authors:** Emma F. Harding, Alice G. Russo, Grace J. H. Yan, Lewis K. Mercer, Peter A. White

**Affiliations:** 1 School of Biotechnology and Biomolecular Sciences, UNSW Sydney, Kensington, NSW Australia; 2 Present Address: Garvan Institute of Medical Research and the Kinghorn Cancer Centre, Cancer Division, Sydney, NSW 2010 Australia

**Keywords:** Metagenomics, Transcriptomics, Next-generation sequencing, Conservation biology

## Abstract

Amphibians and non-avian reptiles represent a significant proportion of terrestrial vertebrates, however knowledge of their viruses is not proportional to their abundance. Many amphibians and reptiles have strict habitual environments and localised populations and are vulnerable to viral outbreaks and potential elimination as a result. We sought to identify viruses that were hidden in amphibian and reptile metatranscriptomic data by screening 235 RNA-sequencing datasets from a 122 species covering 25 countries. We identified 26 novel viruses and eight previously characterised viruses from fifteen different viral families. Twenty-five viruses had RNA genomes with identity to *Arteriviridae*, *Tobaniviridae, Hantaviridae, Rhabdoviridae, Astroviridae, Arenaviridae, Hepeviridae, Picornaviridae, Orthomyxoviridae, Reoviridae, Flaviviridae* and *Caliciviridae*. In addition to RNA viruses, we also screened datasets for DNA viral transcripts, which are commonly excluded from transcriptomic analysis. We identified ten DNA viruses with identity to *Papillomaviridae, Parvoviridae, Circoviridae* and *Adomaviridae*. With the addition of these viruses, we expand the global amphibian and reptile virome and identify new potentially pathogenic viruses that could challenge populations. We speculate that amphibian viruses often have simpler genomes than those in amniotes, as in the case of the *Secondpapillomavirinae* and *Orthomyxoviridae* viruses identified in this study. In addition, we find evidence of inter-family recombination in RNA viruses, and we also identify new members of the recombinant *Adomaviridae* family. Overall, we provide insights into the uncharacterised diversity of amphibian and reptile viruses with the aim of improving population management, treatment and conservation into the future.

## Introduction

Amphibians and non-avian reptiles (herein referred to as reptiles) are ancient lineages of ectotherms, estimated to have diverged from mammals 300–350 million years ago [1]. Since then, they have radiated into over 19,000 species inhabiting every continent except Antarctica [2, 3]. Despite their extensive diversity, there is a considerable gap between genetic knowledge of these animals and that of “higher order” endothermic vertebrates such as mammals and birds. This inequity of available data is illustrated in genome assemblies: 101 reptilian and 39 amphibian genomes are available on NCBI, compared to 735 avian and 2302 mammalian genomes [4, 5]. This lack of knowledge is extended to the paucity of information on amphibian and reptile viruses. Whilst the development and uptake of high throughput sequencing has begun to address this [6, 7], there is still a substantial lack of information about the range of viruses and other pathogens that commonly circulate in amphibians and reptiles.

Viruses are of particular concern to amphibian and reptile populations due to their rapid mutation and evolution rates, resulting in unparalleled diversity and disease capability [8]. During disease outbreaks, viruses can rapidly spread through animal populations with high mortality. Viral discovery is invaluable to understanding the pathogenic challenges these populations face and could facilitate pandemic preparedness by identifying potentially dangerous viruses.

When considering viral threats to amphibians, ranaviruses, large, enveloped DNA viruses, are considered one of the major ecological factors contributing to global population declines [9]. Nidoviruses, medium-large RNA viruses, are also frequently associated with high-mortality outbreaks in reptiles, for example the Bellinger River turtle was almost rendered extinct in 2015 due to an outbreak likely caused by Bellinger River virus [10, 11]. Parvoviruses and circoviruses, small DNA viruses, have lethal associations in reptiles and fish, and were isolated from bearded dragons following mortality events in 2014 and 2020 [12]. Viral discovery can also improve knowledge of zoonotic reservoirs for pathogens and can help assess animal to human spill-over risks based on geographic location. Reptiles have been implicated as reservoirs of arboviral disease, including West Nile virus and Eastern equine encephalitis virus [13]. By studying circulating viruses in amphibians and reptiles we can better understand both the burden of viruses on these hosts and identify viruses with the potential to host-switch within an ecosystem.

Prior to the popularity of next-generation sequencing, knowledge of amphibian and reptile RNA viruses was restricted to the *Paramyxoviridae*, *Retroviridae, Caliciviridae, Togaviridae, Picornaviridae, Flaviviridae* and *Reoviridae* families, only seven of the >150 families that now exist [14]. Since then, representatives from viral families including *Coronaviridae, Astroviridae, Tobaniviridae, Rhabdoviridae, Bornaviridae, Hantaviridae, Hepeviridae, Arteriviridae* and *Arenaviridae* were found to infect reptiles and amphibians [6]. The identification of these novel viruses greatly highlights the unsampled diversity of viruses yet to be found, and the scope of pathogens that could threaten these species. Knowledge of viruses can aid diagnosis of disease outbreaks and assists the conservation of threatened populations. Studying the host range and genetic organisation of novel viruses can also inform us about the evolutionary mechanisms underpinning the evolution of modern viral families. This information is useful for predicting potential future viral disease outbreaks and designing targeted preventative measures.

This study aimed to identify and characterise novel DNA and RNA viruses from amphibian and reptile RNA-Sequencing datasets using a high-throughput viral discovery screen. Two hundred and thirty five publicly available datasets representing a geographically and taxonomically diverse selection of species were used to encompass the diversity of amphibians and reptiles. We also looked for evolutionary insights into these novel viruses using phylogenetic analysis and identifying evidence of recombination.

## Materials and methods

### Selection and assembly of RNA-Seq datasets from amphibians and reptiles

**Fig. 1:**
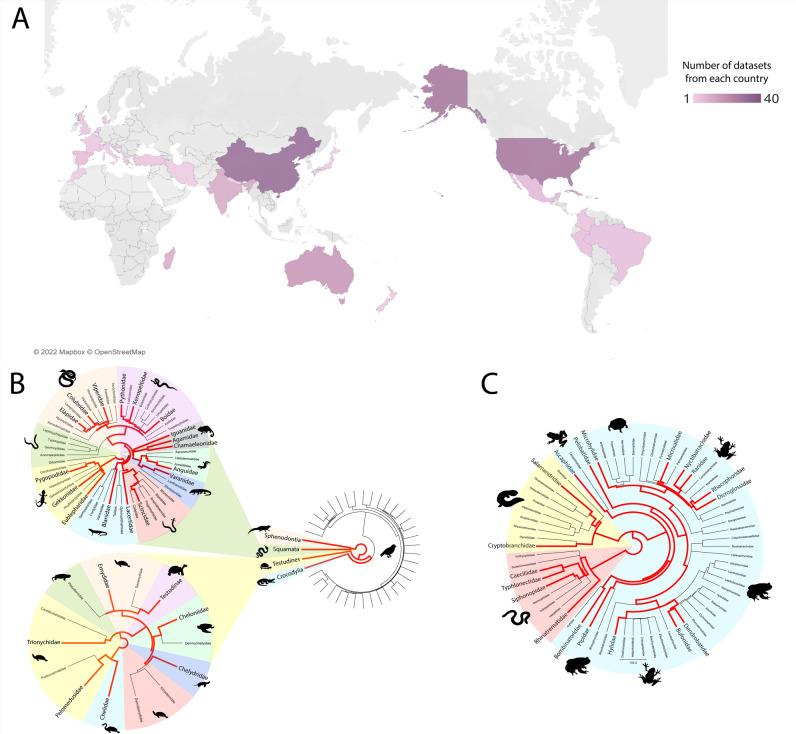
Amphibian and reptile datasets included in this study. **A** geographic location of sample collection. Location was obtained from NCBI SRA biosample records or the associated manuscript, where possible. Datasets where location could not be determined (*n* = 24) were not included in this figure. Colours represent the number of datasets from each location, with dark purple the most and light purple the least. Figure was created using Tableau Desktop (Version 2021.4.4). **B** Taxonomy of saurian datasets. Branches coloured red indicate one or more reptile datasets were obtained from that taxon. Evolutionary trees were generated using TimeTree. **C** Taxonomy of amphibian datasets. Branches coloured red indicate one or more amphibian datasets were obtained from that taxon. Evolutionary trees were generated using TimeTree.

Raw sequencing reads (*n* = 235) encompassing a wide diversity of reptile and amphibian species (*n* = 122) were assembled into transcriptomes for each dataset prior to viral discovery, as previously described [15]. Briefly, publicly available RNA sequencing datasets were obtained from the National Centre of Biotechnology Institute (NCBI) Sequence Read Archive (SRA). Preference was given to datasets using the Illumina HiSeq 2000 platform or newer with no PolyA selection during library preparation, however exceptions were made to include species with minimal available data. Where possible, datasets from each amphibian and reptilian evolutionary clade were selected (Fig. 1). Poor quality reads were trimmed using Trimmomatic prior to *de novo* transcriptome assembly with Trinity version 2.5.1 [16].

### Identification of viral transcripts from assembled datasets

Viral transcripts were distinguished from host cellular and microbiome sequences using a series of increasingly refined BLAST searches, previously described in [17]. Initial annotation was conducted using DIAMOND (v0.9.10) [18] to identify transcripts with identity to viruses from the NCBI non-redundant protein database. These transcripts were imported into Geneious Prime (v2020.1.2) and a further BLASTx search (E-value 1e^−03^) was used to identify and remove transcripts with chance homology to eukaryotic genes and to classify the novel sequences. A combination of BLASTx, tBLASTx and BLASTn searches (E-value 1e^−02^) using genomes of related amphibian and reptilian viruses were conducted on each transcriptome containing a novel virus to locate additional viral transcripts.

We identified an abundance of viral transcripts (>50%) derived from the *Retroviridae*, however many were truncated and contained premature stop codons. Due to the difficult nature of distinguishing exogenous infection from endogenous transcripts, all transcripts with identity to *Retroviridae* genes were excluded from further analysis in this study. Transcripts with identity to plant or invertebrate viruses were also excluded from further analysis, as they were unlikely to be infecting the vertebrate host.

### Viral genome annotation

Annotation of Open Reading Frames (ORFs) and genomic features were conducted in Geneious Prime using comparison against proteins from similar viruses and conserved protein domains. ORFs on each viral transcript were identified using the “Find ORFs” function in Geneious Prime with a minimum length of 200 AA. Each ORF was annotated using a BLASTx search (E-value cut-off 1e^−03^) to determine the identity of the encoded viral protein based on the closest relative. The viral transcripts were also queried against the NCBI conserved domain database (CDD) to facilitate annotation of protein domains and motifs.

### Phylogenetic analysis

To infer phylogeny of novel viruses, viral transcripts translated *in silico* were aligned with proteins from related viruses using MAFFT (v7.407) [19] with the local pair parameter. Related viruses were selected using the closest BLASTx hit for each transcript and downloaded from the NCBI RefSeq database. Where possible, conserved non-structural genes were selected for phylogenetics, however these genes were not always detectable in datasets. Phylogenetic trees were inferred using RAxML (v8.2.12) [20] with the PROTGAMMAAUTO model parameter and 500 bootstrap replicates.

## Results

### An overview of the amphibian and reptile virome

**Fig. 2:**
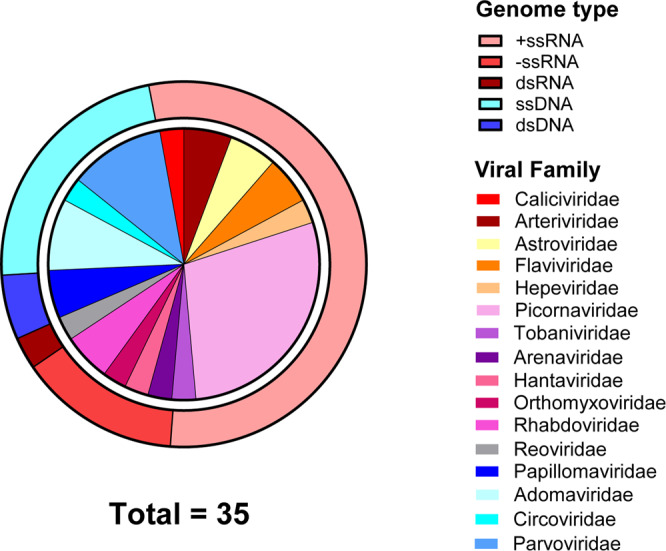
Viruses identified in amphibian and reptile transcriptomes. Viral transcripts, representing 35 viruses were identified using DIAMOND and confirmed with a reciprocal BLASTx search against the NCBI nr database to confirm viral similarity. Outer circle shows the genome type of the novel viruses, and the inner circle shows the viral family classification based on phylogenetic analysis.

We expanded the known virome of amphibians and reptiles by the discovery of 26 novel viruses. In addition, we expanded or confirmed the host range for eight previously reported viruses. A total of 235 RNA-Sequencing datasets from 79 amphibians and 156 reptiles from a variety of geographic locations were screened to identify novel viruses associated with these hosts (Fig. 1, Supplementary Table 1 and Supplementary Figs. 1–5). We identified 26 novel viruses, defined as sharing <90% nucleotide identity with a previously characterised virus (over the contig lengths), and a further nine previously recorded viruses (Table 1). Of the 35 viruses, 15 viral families were represented: 11 with RNA genomes and four with DNA genomes (Table 1 and Fig. 2).

**Table 1:** Viruses identified in amphibian and reptile RNA-Sequencing datasets.

Virus	Family	Animals	Tissue	Dataset	Location	Genome	Presumptive Host	Genome coverage	Novel	Contig length/s (nt)	Identity to closest relative (nt %)
Anole arenavirus	*Arenaviridae*	*Anolis allogus*	Liver	DRR055064	Cuba	-ssRNA	*Sauria*	Partial	Yes	2055, 1495	54.7, 51.8
Chinese broad-headed pond turtle arterivirus	*Arteriviridae*	*Mauremys reevesii*	Mixed viscera	SRR2980465	China	+ssRNA	*Sauria*	NA	No	–	–
Pond slider nidovirus	*Arteriviridae*	*Trachemys scripta*	Liver	ERR2198830	Denmark	+ssRNA	*Sauria*	Partial	Yes	17393	55.7
Gecko astrovirus	*Astroviridae*	*Strophurus ciliaris*	Liver	SRR3901729	Australia	+ssRNA	*Sauria*	Partial	Yes	1852	54.4
Newt calicivirus	*Caliciviridae*	*Cynops pyrrhogaster*	Mixed viscera	SRR1553357	Japan	+ssRNA	*Amphibia*	Full	Yes	7390	65.5
Gecko hepacivirus	*Flaviviridae*	*Oedura marmorata*	Liver	SRR3901728	Australia	+ssRNA	*Sauria*	Partial	Yes	280	55.4
Pond slider hepacivirus	*Flaviviridae*	*Trachemys scripta*	Liver	ERR2198830	Denmark	+ssRNA	*Sauria*	Partial	Yes	1042, 1678, 506, 339	57.9, 58.5, 59.7, 60.3
Skink hantavirus	*Hantaviridae*	*Scincilla lateralis*	Liver	SRR629642	United States	-ssRNA	*Sauria*	Partial	Yes	2274, 909	55.8, 58.5
Red-eared slider hepevirus	*Hepeviridae*	*Trachemys scripta elegans*	Liver	SRR7540570	China	+ssRNA	*Sauria*	Partial	Yes	2476	50
Newt hepevirus	*Hepeviridae*	*Cynops pyrrhogaster*	Mixed viscera	SRR1553357	Japan	+ssRNA	*Amphibia*	Partial	Yes	4658	50
Newt influenza virus	*Orthomyxoviridae*	*Cynops pyrrhogaster*	Mixed viscera	SRR1553357	Japan	-ssRNA	*Amphibia*	Partial	Yes	1824, 1484, 1977, 2156, 2341, 2395	58.4, 60.5, 58.3, 66.7, 72.1, 66.2
Pelodiscus sinsensis picornavirus 1	*Picornaviridae*	*Pelodiscus sinsensis*	Liver	SRR6180862, SRR6180863, SRR6180868, SRR6180867, SRR6157006, SRR6180865, SRR6180859, SRR6180866	China	+ssRNA	*Sauria*	NA	No	–	–
Pelodiscus sinsensis picornavirus 2	*Picornaviridae*	*Pelodiscus sinsensis*	Liver	SRR7540569	China	+ssRNA	*Sauria*	NA	No	–	–
Chameleon picornavirus 1	*Picornaviridae*	*Kinyongia boehmei,Trioceros hoehnelii, Rhampholeon acuminatus, Podarcis muralis, Timon pater*	Mixed viscera	SRR9298917, SRR9298918, SRR9298919, SRR9090248, SRR9090240	Madagascar, Europe	+ssRNA	*Sauria*	Full	Yes	7917	51.2
Chameleon picornavirus 2	*Picornaviridae*	*Brookesia superciliaris*	Mixed viscera	SRR9298920	Madagascar	+ssRNA	*Sauria*	Partial	Yes	293, 1146	75.8, 60.8
Worm lizard picornavirus	*Picornaviridae*	*Blanus cinereus*	Mixed viscera	SRR9090244	Spain	+ssRNA	*Sauria*	Full	Yes	8101	55.3
Omei lazy toad picornavirus	*Picornaviridae*	*Oreolalax omeimontis*	Mixed viscera	SRR8991298	China	+ssRNA	*Amphibia*	Partial	Yes	507, 1455	54.0, 55.2
Red-eared slider pemapivirus	*Picornaviridae*	*Trachemys scripta elegans*	Liver	SRR7540570	China	+ssRNA	*Sauria*	Partial	Yes	2946	74.6
Tremovirus B2	*Picornaviridae*	*Trachemys scripta elegans*	Liver	SRR7540570	China	+ssRNA	*Sauria*	Partial	No	–	–
Red-eared slider tremovirus 1	*Picornaviridae*	*Trachemys scripta elegans*	Liver	SRR7540569	China	+ssRNA	*Sauria*	Partial	Yes	5291, 844	89.8
Red-eared slider picornavirus	*Picornaviridae*	*Trachemys scripta elegans*	Liver	SRR7540569	China	+ssRNA	*Sauria*	Partial	No	–	–
Bush viper reovirus	*Reoviridae*	*Gloydius intermedius*	Mixed viscera	SRR8272683	Asia	dsRNA	*Sauria*	NA	No	–	–
Anole lyssa-like virus	*Rhabdoviridae*	*Anolis allogus*	Liver	DRR055065, DRR055061	Cuba	-ssRNA	*Sauria*	Partial	No	–	–
Rabies lyssavirus	*Rhabdoviridae*	*Alligator sinensis*	Kidney	SRR4212880	China	-ssRNA	*Sauria*	Partial	No	264, 319, 1203, 882	100, 100, 99.9, 99.7
Chameleon serpentovirus	*Tobaniviridae*	*Kinyongia boehmei*	Mixed viscera	SRR9298917	Madagascar	+ssRNA	*Sauria*	Partial	Yes	8120	57.5
Caecilian adomavirus 1	*Adomaviridae*	*Microcaecilia unicolor*	Lung	SRR5591424	French Guiana	ssDNA	*Amphibia*	Partial	Yes	329, 359, 286	55.1, 59.9, 61.4
Caecilian adomavirus 2	*Adomaviridae*	*Typhlonectes compressicauda*	Lung	SRR5591417	French Guiana	ssDNA	*Amphibia*	Partial	Yes	265	57.1
Skink adomavirus	*Adomaviridae*	*Scincilla lateralis*	Liver	SRR629642	United States	ssDNA	*Sauria*	Partial	Yes	917	65.3
Caecilian circovirus	*Circoviridae*	*Caecilia tentaculata*	Testis, skin	SRR5591445, SRR5591449	French Guiana	ssDNA	*Amphibia*	Partial	Yes	983	56.2
Bearded dragon parvovirus	*Parvoviridae*	*Pogona vitticeps*	Heart	SRR8925843, SRR8925842	Australia	ssDNA	*Sauria*	NA	No	–	–
Garter snake parvovirus	*Parvoviridae*	*Thamnophis elegans*	Liver	SRR497737, SRR497744	United States	ssDNA	*Sauria*	Partial	Yes	2795	62.4
Leopard lizard parvovirus	*Parvoviridae*	*Gambelia wislizenii*	Liver, Kidney	SRR7830697, SRR7830700	Mexico	ssDNA	*Sauria*	Partial	Yes	2519	67.4
Red-eared slider parvovirus	*Parvoviridae*	*Trachemys scripta elegans*	Liver	SRR7540571	China	ssDNA	*Sauria*	Partial	Yes	2307	53.6
Archaeolacerta bedriagae papillomavirus	*Papillomaviridae*	*Archaeolacerta bedriagae*	Mixed viscera	SRR9090253	France	dsDNA	*Sauria*	Partial	Yes	2557	50.7
Oreolalax rhodostigmatus papillomavirus	*Papillomaviridae*	*Oreolalax rhodostigmatus*	Mixed viscera	SRR8991300	China	dsDNA	*Sauria*	Partial	Yes	1130, 1420	57.3, 51.5

Italic text denotes viral family names, scientific names for animals, and presumptive host (the class of each host).

Positive-sense single-stranded RNA (+ssRNA) viruses were the most abundant genome type (54%) with ten of the 35 viruses sharing identity to *Picornaviridae* viruses (Fig. 2). Aside from picornaviruses, viruses from other RNA viral families were detected included nine with +ssRNA genomes, five with -ssRNA genomes and one with a dsRNA genome (Table 1 and Fig. 2). Novel RNA viruses had identity to *Astroviridae, Hepeviridae, Hantaviridae, Arenaviridae, Tobaniviridae, Flaviviridae, Arteriviridae, Orthomyxoviridae* and *Caliciviridae* viruses (Table 1 and Fig. 2).

### Picornaviruses are prevalent globally

**Fig. 3:**
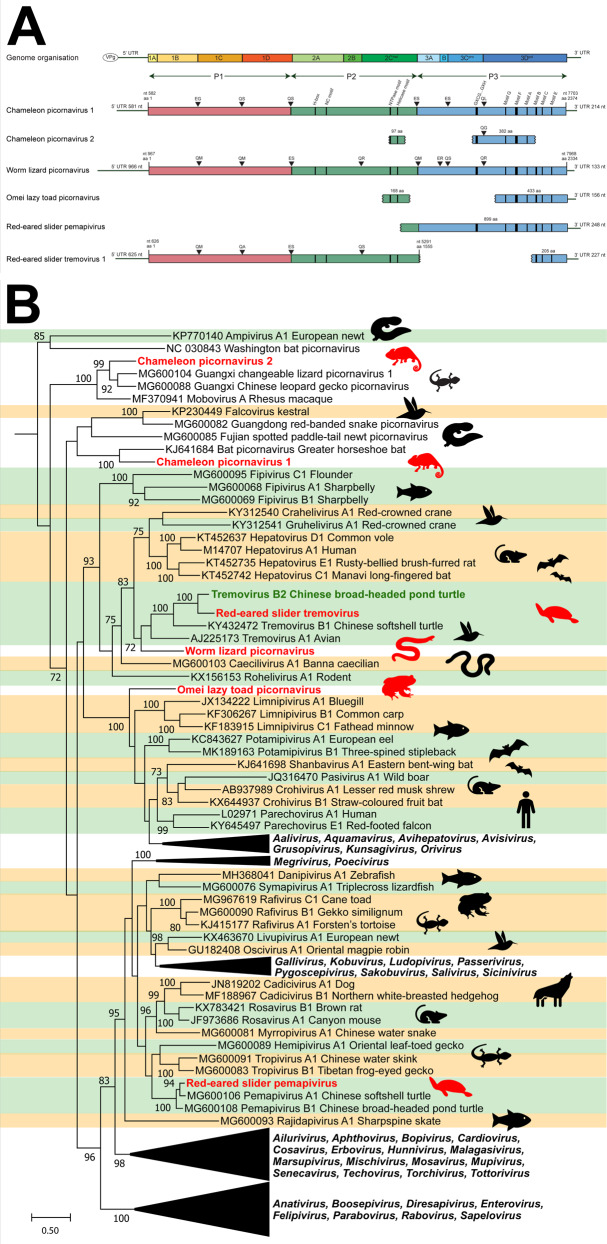
Novel picornaviruses identified in amphibians and reptiles. Contigs were identified using a BLAST search of annotated picornavirus proteins downloaded from NCBI protein database. **A** Genome organisation of novel picornaviruses. ORFs were annotated using Geneious Prime 2020.1, and motifs were identified based on literature. Protease cleavage sites were predicted based on preferred cleavage templates and estimated size of mature proteins. Estimated viral segments are colour-coded with P1 in purple, P2 in blue and P3 in orange. Genome position (nt) is indicated by the black bar along the bottom. **B** Phylogeny of novel picornaviruses. The 3D polymerase region of novel picornaviruses was *in silico* translated and aligned with reference picornaviruses using MAFFT. Phylogenetic trees were constructed using RAxML with 500 bootstrap replicates. Novel viruses are red, previously discovered viruses are green and known genera are shaded alternating green and orange. The scale bar represents substitutions per site.

Picornaviruses were the most prevalent viruses identified in the amphibian and reptile species examined (*n* = 10/35) (Table 1 and Fig. 3). We discovered two full-length picornavirus genomes and eight partial genomes (ranging from 1 439-6 135 nt coverage) (Table 1).

The two full-length novel picornaviruses were found in five lizard species (Table 1). Specifically, chameleon picornavirus 1 had a 7122 kb ORF with a 5′ UTR of 581 nt and a 3′ UTR of 215 nt (Fig. 3, Panel A). The virus was present in mixed viscera from three chameleon species in Madagascar: Van Höhnel’s chameleon (*Trioceros hoehnelii*), Bohme’s two horned chameleon (*Kinyongia boehmei*) and the Nguru pygmy chameleon (*Rhampholeon acuminatus*) (Table 1). Chameleon picornavirus 1 was also identified in two wall lizards from Northern Africa and Europe (*Timon pater* and *Podarcis muralis*).

Worm lizard picornavirus had a 7002 kb ORF with a 5′UTR of 967 nt and a 3′UTR of 134 nt and was detected in mixed viscera from a European worm lizard (*Blanus cinereus*) from Spain (Fig. 3, Panel A).

Eight partial picornavirus genomes were also identified, four of which represented novel viruses. These four included chameleon picornavirus 2 from a brown leaf chameleon (*Brookesia superciliaris*) in Madagascar, Omei lazy toad picornavirus from a toad (*Oreolalax omeimontis*) from China, red-eared slider tremovirus and red-eared slider pemapivirus from turtles (*Trachemys scripta elegans*) from China (Table 1).

To further classify the novel picornaviruses, we inferred a maximum likelihood (ML) phylogeny of the 3D RNA-dependent RNA-polymerase region with reference sequences from each known picornavirus genus and newly discovered, unclassified representatives. This revealed the distribution of novel picornaviruses amongst the known genera (Fig. 3, Panel B). Chameleon picornavirus 1 and 2 clustered with unclassified reptile and amphibian picornaviruses, whilst worm lizard picornavirus and red-eared slider tremovirus clustered near turtle and bird tremoviruses. The Omei lazy toad picornavirus clustered near fish limnipivuruses, and the red-eared slider pemapivirus clustered near other turtle pemapiviruses (Fig. 3, Panel B).

### Two nidoviruses with divergent structural genes

**Fig. 4:**
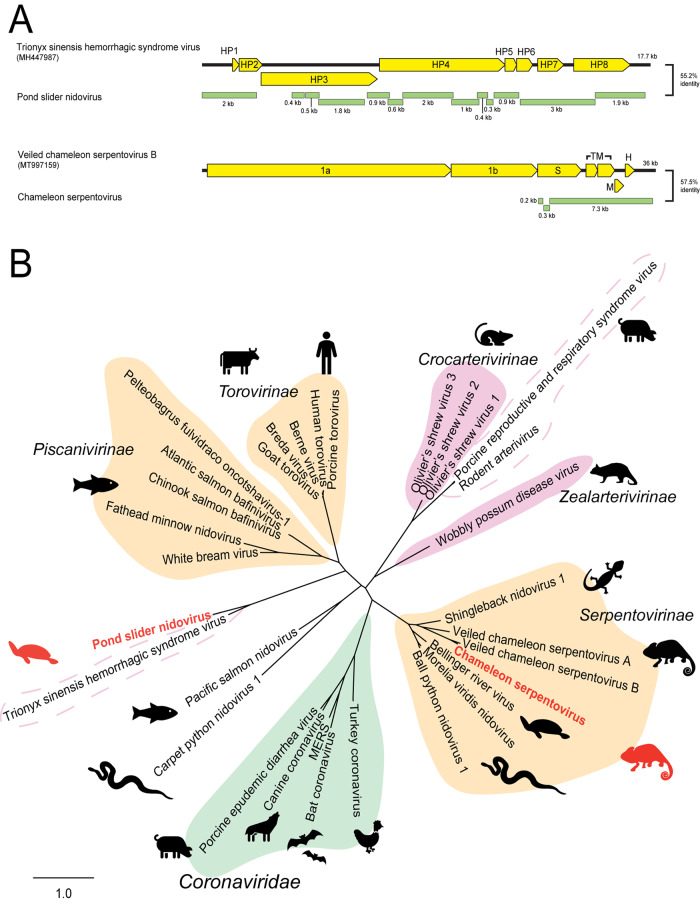
Novel nidoviruses in reptiles. Contigs were identified using a BLAST search of annotated nidovirus proteins downloaded from NCBI protein database against transcriptomes. **A** Genome organisation of novel nidoviruses. Viral contigs (green) were aligned with reference genomes (yellow) using MAFFT. **B** Phylogeny of nidoviruses. The membrane/HP8 gene of nidoviruses were *in silico* translated and 170 AA was aligned with reference viruses using MAFFT. Phylogenetic trees were constructed using RAxML with 500 bootstrap replicates. Novel viruses are red and known classifications are shaded as follows: Orange:*Tobaniviridae*, Green:*Coronaviridae*, Pink:*Arteriviridae*. Dotted outlines indicate viruses with no assigned genus within the *Arteriviridae* family. The scale bar represents substitutions per site.

Two novel RNA *Nidovirales* were identified in this study in a pond slider turtle (*Trachemys scripta*) from a farm in Denmark and a chameleon (*Kinyongia boehmei*) from Madagascar (Table 1 and Fig. 4).

The non-structural proteins of Pond slider nidovirus were related to nidovirus proteins, however we found six structural ORFs with no viral homologue within the *Nidovirales*, suggesting this virus has very different structural characteristics. The Pond slider nidovirus contained eight ORFs with nucleotide identity (51–68%) to hypothetical genes 1–8 in Trionyx sinensis hemorrhagic syndrome virus (TSHSV)(NCBI: MH447967)(Fig. 4, Panel A). When translated, Pond slider nidovirus proteins shared 28–53% AA identity to the equivalent proteins in TSHSV. Hypothetical protein 8 (putative membrane protein) phylogeny placed Pond slider arterivirus in a distinct clade with TSHSV of unclassified *Arteriviridae* (Fig. 4, Panel B).

The chameleon serpentovirus contig contained five predicted structural ORFs, of which only two had related proteins through BLAST searches: ORF2 which encodes a spike glycoprotein and ORF4 which encodes a membrane protein. Phylogenetic analysis of the membrane gene clustered chameleon serpentovirus within the *Serpentovirinae* subfamily of *Tobaniviridae* near other reptilian viruses (Fig. 4, Panel B).

### Novel hepeviruses and astroviruses confirm the family’s recombinant origin

The *Hepeviridae* are a family of RNA viruses resulting from a recombination event between the structural regions (capsid) of *Astroviridae* and the non-structural regions (ORF1) of *Alphatetroviridae-*like viruses [21]. Three viruses with identity to *Hepeviridae* and *Astroviridae* were present in a newt (*Cynops pyrrhogaster*) from Japan, a turtle (*Trachemys scripta elegans)* from China and a gecko (*Strophurus ciliaris*) from Australia (Table 1 and Supplementary Fig. 3).

Newt hepevirus had ORF1 identity (52% over 2 506 nt) to Orthohepevirus A (NCBI: NC_001434) and capsid identity (50% over 2 226 nt) to cutthroat trout virus (NCBI: NC_015521). Red-eared slider hepevirus had ORF1 identity (52% over 622 nt) to bat hepevirus (NCBI: LC340969) and capsid identity (50% over 1854 nt) to mamastrovirus sp. (NCBI: MW826530). Only the capsid of gecko astrovirus was detectable, and it shared 54% identity over 1852 nt with Zhejiang chinese fire belly newt astrovirus 2 (NCBI: MG599916) (Supplementary Fig. 3).

### Rabies lyssavirus in an alligator

An alligator (*Alligator sinensis*) kidney dataset from China contained contigs with >99% nt identity to rabies lyssavirus genes (NCBI: NC_001542) (Table 1). We identified contigs covering parts of the nucleoprotein, phosphoprotein, M2 and glycoprotein genes (Supplementary Figure 2). The nucleotide identity of the alligator-associated virus nucleoprotein was 99.9% compared to that of Rabies lyssavirus (Table 1), indicating it is rabies lyssavirus [14]. The phylogeny groupings of the nucleoprotein confirmed this classification (Supplementary Fig. 2).

### An amphibian influenza A virus

Mammalian and avian influenza viruses are RNA viruses with 8–10 distinct segments [7]. We discovered six genomic segments from a novel influenza virus in a Japanese fire belly newt (*Cynops pyrrhogaster*) (Table 1). These six segments had full-length identity to the six segments of Wuhan Asiatic toad influenza virus (NCBI: MG600045-50) representing the genes: PA (67% nt identity), PB1 (72% nt identity), PB2 (66% identity), HA (59% nt identity), NA (60% identity) and NP (58% identity) (Table 1). Newt influenza virus phylogenetically clustered basally to endotherm influenza A viruses (Supplementary Fig. 4).

### DNA viruses detected from RNA-Sequencing data

We used a BLAST-based bioinformatics workflow to identify RNA transcripts from novel DNA viruses in metatranscriptomic data. We identified contigs from ten distinct DNA viruses with identity to *Parvoviridae* (*n* = 4), *Circoviridae* (*n* = 1), *Papillomaviridae* (*n* = 2) and *Adomaviridae* (*n* = 3) families (Table 1).

**Fig. 5:**
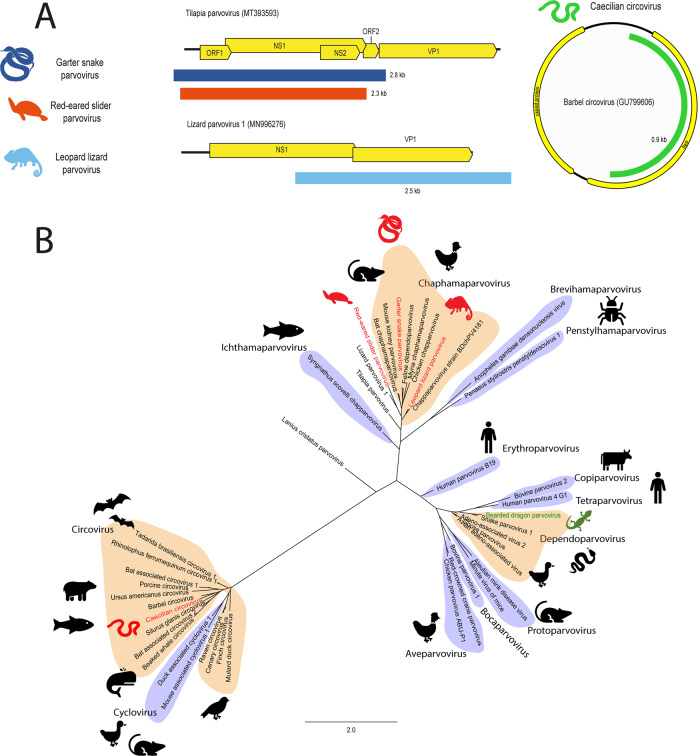
Novel parvovirus and circovirus identified in amphibians and reptiles. Contigs were identified using a BLAST search of annotated parvovirus and circovirus proteins downloaded from NCBI protein database against transcriptomes. **A** Genome organisation of novel parvoviruses and circovirus. Viral contigs were aligned with reference genomes using MAFFT. Contigs are coloured based on organism of origin**. B** Phylogeny of parvoviruses and circoviruses. The NS1 region of parvovirus and circoviruses were *in silico* translated and 131 AA was aligned with reference viruses using MAFFT. Phylogenetic trees were constructed using RAxML with 500 bootstrap replicates. Novel viruses are red and previously discovered viruses are green. Known genera are shaded alternating orange and purple. The scale bar represents substitutions per site.

Contigs that represent four partial genomes (range 2.3–2.8 kb) of parvoviruses were identified from two lizards, one turtle and a snake including the Western terrestrial garter snake (*Thamnophis elegans*) from the USA, the red-eared slider (*Trachemys scripta elegans*) from China, the long-nosed leopard lizard (*Gambelia wislizenii*) from Mexico and the central bearded dragon (*Pogona vitticeps*) from Australia (Table 1 and Fig. 5).

The three novel parvoviruses; red-eared slider parvovirus, leopard lizard parvovirus and garter snake parvovirus, phylogenetically cluster within the *Chaphamaparvovirus* genus near previously identified viruses infecting lizards, birds and mammals (Fig. 5, Panel B). These novel viruses shared 53–67% pairwise identity over 2307-2795 nt to other chaphamaparvoviruses (Table 1). The central bearded dragon parvovirus sequences were closely related to the bearded dragon parvovirus (NCBI Accession: NC_027429) of the *Dependoparvovirus* genus and shared 94–99% nt identity across the identified transcripts, indicating it is likely to be the same virus.

The partial genome of a novel circovirus was constructed from two transcripts (0.3 and 0.7 kb, total 983 nt) identified in a caecilian (*Caecilia tentaculate*) from French Guiana (Table 1 and Fig. 5, Panel A). Caecilian circovirus phylogenetically clustered in a clade with fish-infecting viruses within the *Circovirus* genus (Fig. 5, Panel B) and the closest relative was Barbel circovirus (NCBI Accession: GU799606) from Hungary, with 56% over 825 nt of the NS1 gene (Table 1).

### Novel members of the recombinant Adomaviridae family

**Fig. 6:**
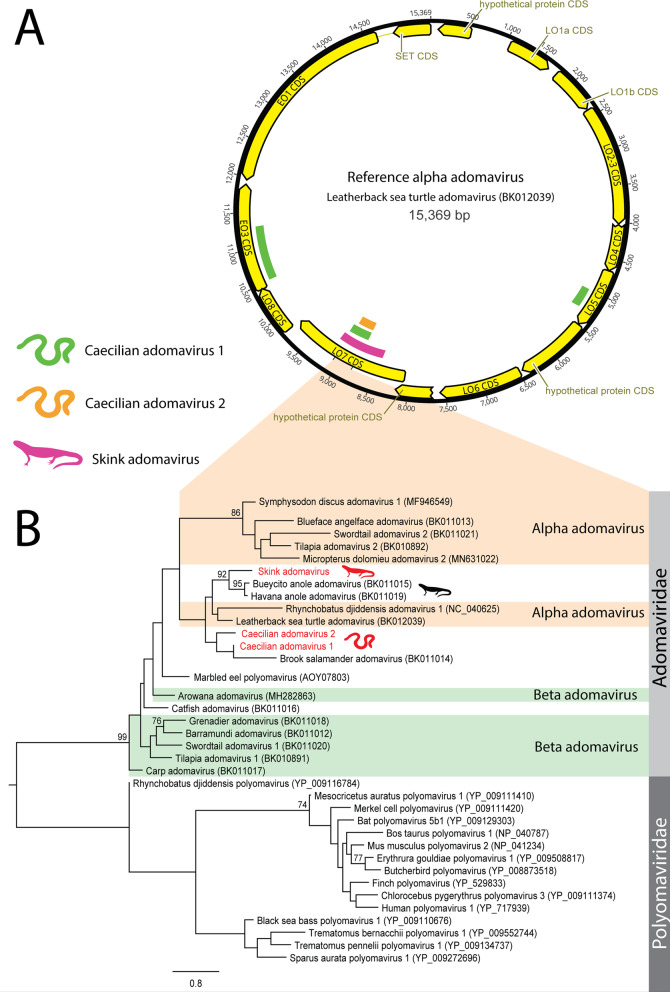
Adomavirus-like contigs identified in amphibians and reptiles. **A** Transcripts were identified using BLAST searches of annotated adomavirus proteins downloaded from NCBI protein database against RNA-Sequencing datasets. **B** Phylogenetic analysis was conducted on 87 AA of the in silico translated LO7 contigs. Adomavirus contigs were aligned using MAFFT and phylogenetic trees were constructed using RAxML with 500 bootstrap replicates. Proposed adomavirus classifications are denoted with orange and green shading. The scale bar represents substitutions per site.

Adomaviruses are as yet an unconfirmed family of DNA viruses of suspected recombinant origin, and share similar genes with papillomaviruses, polyomaviruses and adenoviruses [11]. We identified six contigs (range = 265–917 nt) from three viruses with identity to adomaviral genes from a skink (*Scincilla lateralis*) from the USA and two caecilians (*Microcaecilia unicolor* and *Typhlonectes compressicauda*) from French Guiana (Table 1 & Fig. 6). The phylogeny of the three novel adomaviruses was examined using a partial reconstructed late ORF 7 (LO7) aa sequence (87 AA), which clustered the caecilian adomaviruses near leatherback sea turtle adomavirus (NCBI: BK012039, 56–58% identity over 329 nt) and the skink adomavirus with Bueycito anole adomavirus (NCBI: BK11015, 62% identity over 917 nt), both within the alpha adomavirus classification (Fig. 6, Panel B).

### Amphibian and reptile papillomaviruses form distinct clades from mammalian papillomaviruses

**Fig. 7:**
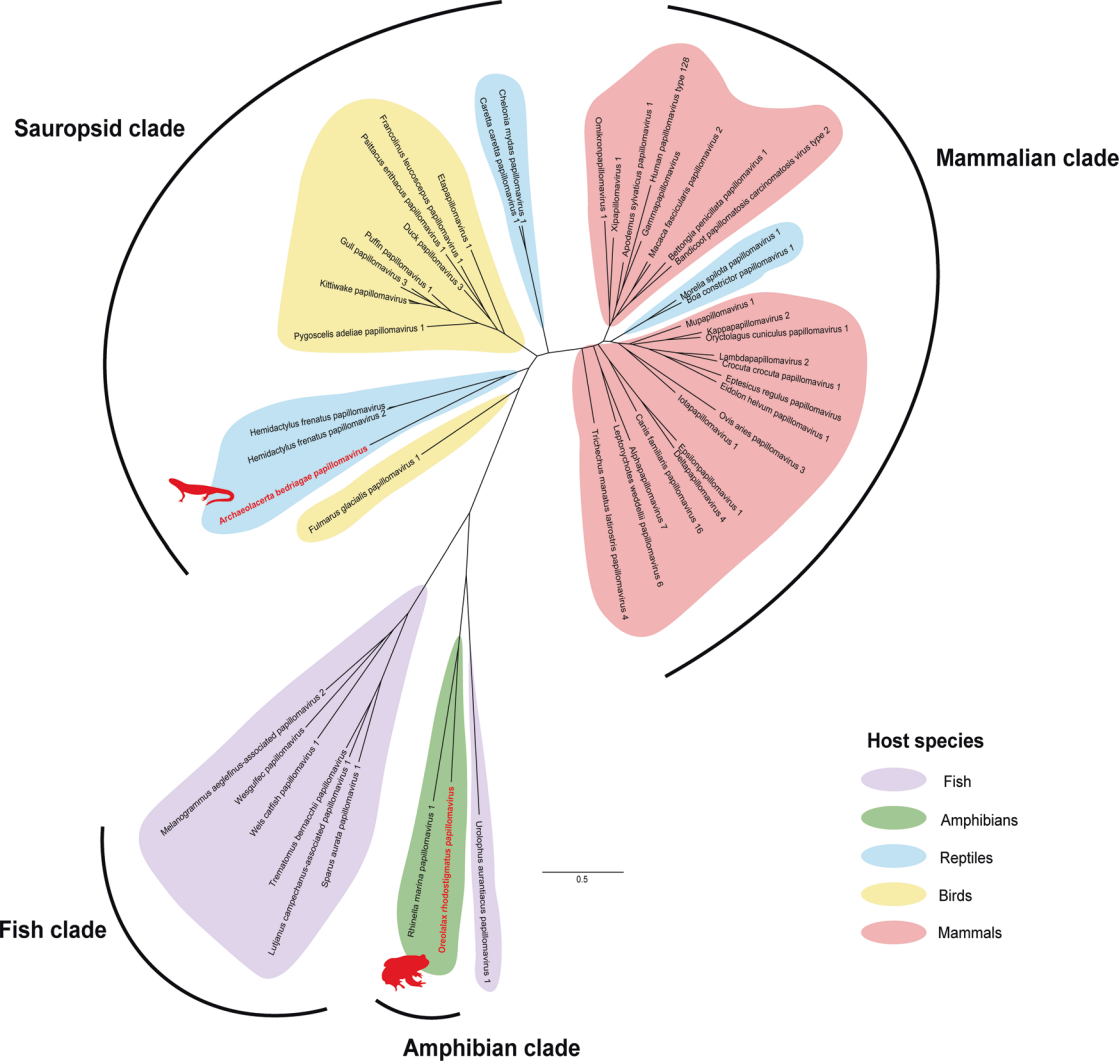
Novel amphibian and reptile papillomaviruses. We identified contigs from two novel papillomaviruses from RNA-Sequencing datasets using BLAST-based methodology. Contigs of the L1 gene (1389 nt) were aligned and *in silico* translated before MAFFT alignment with reference *Papillomaviridae*. The phylogenetic tree was constructed using RAxML with 500 bootstrap replicates. Novel viruses are indicated in red. Phylogenetic clades are shaded based on host: purple for fish, green for amphibians, blue for non-avian reptiles, yellow for birds and red for mammals. The scale bar represents substitutions per site.

Papillomaviruses are double-stranded DNA viruses with genomes ~8 kb [22]. We detected two novel papillomaviruses in datasets from mixed viscera from a Guizhou lazy toad (*Oreolalax rhodostigmatus*) from China and a Bedriaga’s rock lizard (*Archaeolacerta bedriagae*) from Corsica and Sardinia islands in Europe (Table 1). We recovered the full L1 gene (1389 nt) of both viruses and the full E2 gene (801 nt) of Oreolalax rhodostigmatus papillomavirus (Fig. 7).

L1 phylogeny clustered Oreolalax rhodostigmatus papillomavirus within the *Secondpapillomavirinae* as the second amphibian virus discovered in this subfamily (Fig. 7). Oreolalax rhodostigmatus papillomavirus forms a clade with another amphibian papillomavirus: Rhinella marina papillomavirus (NCBI: MW582900) from a cane toad and shared 57% identity over 1130 nt (Table 1).

Archaeolacerta bedriagae papillomavirus clustered within the *Firstpapillomavirinae* subfamily in a distinct clade with Hemidactylus frenatus papillomaviruses of geckos (NCBI: MK207055) and shared 51% identity over 2557 nt (Table 1 and Fig. 7). This clade sits within the grouping of reptilian papillomaviruses including *Dyozetapapillomavirus* in turtles and is located between the *Secondpapillomavirinae* and avian papillomaviruses.

## Discussion

We aimed to expand the amphibian and reptile virome using viral discovery to identify DNA and RNA viruses hidden in metatranscriptomic datasets. We sampled 235 datasets from 25 countries and 122 different species to encompass a diversity of amphibians and reptiles (Fig. 1 and Supplementary Table 1). We identified a total of 26 novel viruses and nine previously identified viruses and expand the known diversity of viruses and our understanding of their evolution.

Many viral families or genera traditionally thought to be exclusively associated with mammals also infect reptiles and amphibians [6, 23, 24]. We identified reptile-associated *Arenaviridae*, and *Lyssavirus* viruses which, until recently, were thought to be endotherm-specific (Table 1) [25, 26]. These reptile viruses are phylogenetically basal to endotherm viruses and have few close relatives (Supplementary Figs. 1 and 4). It is likely that these viruses are the first representatives of much broader clades, genera and subfamilies of viruses which have diversified within reptiles.

### Discovery of DNA viruses from RNA-Sequencing datasets

The discovery of DNA viruses primarily relies on either tissue DNA sequencing or PCR-based approaches. Many new viruses have been discovered this way [27–29], however a novel approach is to utilise transcriptomic data to discover DNA viruses through bioinformatic approaches [15, 30–32].

Studies based on RNA-sequencing normally ignore or fail to identify DNA viruses, however in this study we identify genes from ten DNA viruses through translation of the RNA dataset with subsequent searches for DNA viral homologues. In the case of understudied species such as amphibians and reptiles, obtaining maximal information from limited samples is vital, and the presence of DNA virus transcripts in RNA sequencing datasets should not be overlooked.

### Inter-family viral recombination

Co-infection of viruses is common in amphibians and reptiles and provides frequent opportunities for recombination [12, 33]. Recombination often occurs at the junction of the structural and non-structural genes and has occurred ubiquitously throughout viral evolution [34]. The DNA-RNA chimeric viruses cruciviruses and the plant-animal virus hybrids *Hepeviridae* are two of many viral groups formed through recombination between unrelated viruses [21, 35].

*Adomaviridae* viruses are a recently proposed family believed to be the result of recombination resulting in viruses with adenovirus structural genes and papilloma/polyomavirus non-structural genes [36–38]. Previously identified adomaviruses have been isolated from fish and reptiles coinfected with other small DNA viruses including *Papillomaviridae* and *Polyomaviridae*, providing ample opportunities for genetic mixing [36, 37]. The three novel adomaviruses from this study contained genes with weak (<35% AA over 204 AA) identity to polyomavirus major capsid genes, supporting the shared history of these viral families.

The RNA *Nidovirales* contain several families of viruses with similar genomic structure: one or two long non-structural 5′ ORFs and several shorter 3′ structural ORFs [39]. Both novel nidoviruses discovered herein share this genome layout, however, the structural genes are highly divergent (Fig. 4). Pond slider nidovirus contains eight ORFs: the non-structural ORFs 2 and 3 have identity to other nidovirus 1ab genes, however the predicted structural ORFs 4–8 have no viral homologues other than the unclassified arterivirus TSHSV. The TSHSV genome is approximately 18 kb and codes for eight proteins, seven of which have not been characterised and have unknown functions [40]. The lack of homology between the predicted structural proteins of pond slider nidovirus, TSHSV and other arteriviruses suggests a recombination event at the non-structural/structural junction introduced a novel structural gene combination at the 3′ end of these nidoviruses. Phylogeny of the predicted membrane protein confirms their divergence from other classified *Arteriviridae*, as pond slider nidovirus and TSHSV form a new clade within *Nidovirales* (Fig. 4).

We also identified two novel viruses from the *Hepeviridae*, a viral family of recombinant origins between *Alphatetroviridae-*like non-structural genes and animal *Astroviridae* structural genes [21]. Both newt hepevirus and red-eared slider hepevirus have gene homology consistent with genomic characteristics of *Hepeviridae* and illustrates the wide success of this recombinant family in diversifying throughout taxa into newts and turtles.

### Amphibian viruses often have simple genomes

Many amphibian viruses have simpler genomes with few or no accessory genes compared to endotherm viruses of the same families [7, 32, 41]. These “simpler” viruses often form clades which roughly follow host evolution and phylogenetically cluster as intermediates between fish and amniote viruses (Fig. 7 and Supplementary Figs. 1–4) [6, 32, 42–44].

We identified a novel member of the DNA subfamily of the *Papillomaviridae* family, namely *Secondpapillomavirinae*, which to-date only contains one formally recognised virus [45]. The *Secondpapillomavirinae* viruses comprise greatly condensed viral genomes, conserving only the core E1-E2-L2-L1 genes, which may represent ancestral sequences that diversified in lower vertebrates [30, 33, 46]. Oreolalax rhodostigmatus papillomavirus is the second amphibian papillomavirus discovered, and the newest member of the *Secondpapillomavirinae* subfamily of fish and amphibian papillomaviruses (Fig. 7). It is likely that there is a wide diversity of undiscovered *Secondpapillomavirinae* within fish and amphibians to parallel the diversity of the *Firstpapillomavirinae*.

No amphibian or fish *Firstpapillomavirinae* have been discovered, suggesting *Firstpapillomavirinae* exclusively infect amniotes and diverged from the *Secondpapillomavirinae* with the evolution of saurian and mammalian classes [46, 47]. Supporting the host-specificity of each *Papillomaviridae* subfamily, Archaeolacerta bedriagae papillomavirus from a lizard cluster within the sauropsid clade of *Firstpapillomavirinae* (Fig. 7).

### Using viral discovery to understand potential viral threats to amphibians and reptiles

Until recently, pathogen discovery in amphibians and reptiles was focussed on identifying causative agents and associated pathogens during or after severe disease outbreaks. Bellinger river nidovirus, bearded dragon circovirus and chaphamaparvoviruses were all characterised after severe mortality events in reptiles [10, 12]. Diseases are frequently associated with coinfections and may be attributed to complex interactions between multiple pathogens [48], as in the case of tumours in green turtles [49]. As so little is known about the frequency, distribution and diversity of viruses, it is hard to assess the risk that novel viruses pose. However, understanding the types of viruses that circulate in wild and captive populations is the first step in building a knowledge base for rapid diagnostics during outbreaks. Viruses phylogenetically related to known pathogens including nidoviruses and lyssaviruses from this study can be flagged and studied further to aid in the development of monitoring and prevention strategies to protect vulnerable populations.

RNA nidoviruses are known to cause a range of severe symptoms including respiratory distress and kidney damage in reptiles [10, 11, 23, 50]. These viruses comprise families such as *Coronaviridae, Arteriviridae* and *Tobaniviridae*. The two nidoviruses discovered in this paper, pond slider nidovirus and chameleon serpentovirus phylogenetically cluster close to viruses associated with severe disease in reptiles (Fig. 4) [11]. Specifically, chameleon serpentovirus clusters within the *Serpentovirinae* subfamily of viruses associated with reptile mortality including Bellinger River virus, Morelia viridis nidovirus of pythons and Shingleback nidovirus 1 (Fig. 4) [11, 51–53]. Pond slider nidovirus is closest related to TSHSV, an unclassified arterivirus which causes fatal disease in turtles (Fig. 4) [40]. Whilst the definitive link between viral infection and pathogenesis is under investigation, the association between nidoviruses and reptile disease is well documented [11]. Due to the repeated associations with disease, nidoviruses in reptiles should be extensively studied to better understand the implications of infection and threat they pose to wild populations.

Lyssaviruses have only recently been described in reptiles [25], and their presence in both a skink and an alligator suggests they infect a wider range of saurians than previously documented (Table 1 and Supplementary Fig. 2). Lyssaviruses are neurotropic in mammals and abundant in infected skink brain tissue [25], suggesting they may also have detrimental neurological effects in reptilian hosts. Glycoproteins from reptile lyssaviruses demonstrate a broad animal host range, similar to rabies lyssavirus, indicating they may jump between reptile species and pose a significant viral threat [54].

Additionally, many small DNA viruses are associated with oncogenesis and neoplasia in animals [55–57]. These viruses are well documented in mammals and some birds, however the diversity and pathogenesis in lower vertebrates including fish, reptiles and amphibians have not been extensively studied [7, 58]. This study has identified ten small DNA viruses from families associated with skin lesions and implicated in mortality (Table 1). Several other studies have identified intrusive lesions association with DNA viruses such as circoviruses, parvoviruses, papillomaviruses, polyomaviruses and adomaviruses, highlighting the pressure that DNA virus infection can put on amphibian and reptile hosts [12, 33, 59].

## Conclusion

The global presence and phylogenetic diversity of amphibian and reptile viruses warrant further investigation into their pathogenicity and relative danger to naïve populations. Often the causative or associated viruses in animal disease are identified in response to an outbreak that threatens the population, sometimes several years after the event. This retroactive response is too late to meaningfully contribute to outbreak control or treatment, and the discovery of potential pathogens is essential in transitioning to a proactive monitoring and prevention strategy for vulnerable populations.

In addition, expanding the virome illustrates the history and complexities of vertebrate viruses, including virus-host coevolution and recombination of viral families. We highlight the diversity of amphibian and reptile viruses and show that many are highly divergent from previously characterised viruses. By identifying 26 new amphibian and reptile viruses, we pave the way for future viral discovery studies to uncover the plethora of unknown viruses in ectothermic vertebrates and to inform diagnostic developments.

## Supplementary information

Supplementary Figures 1–5

## Data Availability

All RNA-Sequencing datasets used in this study are publicly available on the NCBI Sequence Read Archive. Nucleotide sequence data of assembled viral contigs are available in the Third Party Annotation Section of the DDBJ/ENA/GenBank databases under the accession numbers TPA: BK061385-BK061397, BK061564-BK061584 and BK062316-BK062321. The Oreolalax rhodostigmatus papillomavirus contig is available on Genbank under the accession MW582921.
