# Supplementary material for: Revealing the uncharacterised diversity of amphibian and reptile viruses
Source: ISME Commun. 2022 Oct 2;2:95. doi: 10.1038/s43705-022-00180-x (PMC9723728; doi:10.1038/s43705-022-00180-x)
Supplement: Supplementary file 1 — Supplementary Figures 1–5 [file 43705_2022_180_MOESM1_ESM.docx]

**Supplementary materials**

Supplementary Table 1 – Datasets selected for viral discovery

Supplementary Figure 1 – Novel reptile *Bunyavirales*

Supplementary Figure 2 – Reptile lyssaviruses

Supplementary Figure 3 – Novel amphibian and reptile *Hepeviridae* and *Astroviridae*

Supplementary Figure 4 – Novel newt influenza virus

Supplementary Figure 5 – Novel newt calicivirus

*Supplementary Table 1:* Publicly available RNA-Sequencing datasets used in this study

| **Species** | **Scientific name** | **Animal type** | **SRA Accession** | **Country of origin** | **Tissue** | **Sequencing platform** |
| --- | --- | --- | --- | --- | --- | --- |
| Cayenne caecilian | *Typhlonectes compressicauda* | Amphibian | SRR5591415 | French Giana | Kidney | Illumina HiSeq2000 |
| Cayenne caecilian | *Typhlonectes compressicauda* | Amphibian | SRR5591416 | French Giana | Heart | Illumina HiSeq2000 |
| Cayenne caecilian | *Typhlonectes compressicauda* | Amphibian | SRR5591417 | French Giana | Lung | Illumina HiSeq2000 |
| Cayenne caecilian | *Typhlonectes compressicauda* | Amphibian | SRR5591418 | French Giana | Liver | Illumina HiSeq2000 |
| Two-lined caecilian | *Rhinatrema bivittatum* | Amphibian | SRR5591419 | French Giana | Skin | Illumina HiSeq2000 |
| Two-lined caecilian | *Rhinatrema bivittatum* | Amphibian | SRR5591420 | French Giana | Liver | Illumina HiSeq2000 |
|  | *Microcaecilia unicolor* | Amphibian | SRR5591421 | French Giana | Skin | Illumina HiSeq2000 |
|  | *Microcaecilia unicolor* | Amphibian | SRR5591422 | French Giana | Posterior skin | Illumina HiSeq2000 |
|  | *Microcaecilia unicolor* | Amphibian | SRR5591423 | French Giana | Muscle | Illumina HiSeq2000 |
|  | *Microcaecilia unicolor* | Amphibian | SRR5591424 | French Giana | Lung | Illumina HiSeq2000 |
|  | *Microcaecilia unicolor* | Amphibian | SRR5591425 | French Giana | Liver | Illumina HiSeq2000 |
|  | *Microcaecilia unicolor* | Amphibian | SRR5591426 | French Giana | Foregut | Illumina HiSeq2000 |
|  | *Microcaecilia dermatophaga* | Amphibian | SRR5591427 | French Giana | Skin | Illumina HiSeq2000 |
|  | *Microcaecilia dermatophaga* | Amphibian | SRR5591428 | French Giana | Posterior skin | Illumina HiSeq2000 |
|  | *Microcaecilia dermatophaga* | Amphibian | SRR5591429 | French Giana | Liver | Illumina HiSeq2000 |
|  | *Microcaecilia dermatophaga* | Amphibian | SRR5591430 | French Giana | Kidney | Illumina HiSeq2000 |
| Cayenne caecilian | *Typhlonectes compressicauda* | Amphibian | SRR5591431 | French Giana | Foregut | Illumina HiSeq2000 |
| Two-lined caecilian | *Rhinatrema bivittatum* | Amphibian | SRR5591432 | French Giana | Testis | Illumina HiSeq2000 |
| Two-lined caecilian | *Rhinatrema bivittatum* | Amphibian | SRR5591433 | French Giana | Muscle | Illumina HiSeq2000 |
| Two-lined caecilian | *Rhinatrema bivittatum* | Amphibian | SRR5591434 | French Giana | Skin | Illumina HiSeq2000 |
| Two-lined caecilian | *Rhinatrema bivittatum* | Amphibian | SRR5591435 | French Giana | Liver | Illumina HiSeq2000 |
| Two-lined caecilian | *Rhinatrema bivittatum* | Amphibian | SRR5591436 | French Giana | Lung | Illumina HiSeq2000 |
|  | *Microcaecilia unicolor* | Amphibian | SRR5591437 | French Giana | Skin | Illumina HiSeq2000 |
|  | *Microcaecilia unicolor* | Amphibian | SRR5591438 | French Giana | Foregut | Illumina HiSeq2000 |
|  | *Microcaecilia unicolor* | Amphibian | SRR5591439 | French Giana | Kidney | Illumina HiSeq2000 |
|  | *Microcaecilia unicolor* | Amphibian | SRR5591440 | French Giana | Liver | Illumina HiSeq2000 |
| Cayenne caecilian | *Typhlonectes compressicauda* | Amphibian | SRR5591441 | French Giana | Skin | Illumina HiSeq2000 |
| Two-lined caecilian | *Rhinatrema bivittatum* | Amphibian | SRR5591442 | French Giana | Spleen | Illumina HiSeq2000 |
| Two-lined caecilian | *Rhinatrema bivittatum* | Amphibian | SRR5591443 | French Giana | Kidney | Illumina HiSeq2000 |
| Cayenne caecilian | *Typhlonectes compressicauda* | Amphibian | SRR5591444 | French Giana | Posterior skin | Illumina HiSeq2000 |
|  | *Caecilia tentaculata* | Amphibian | SRR5591445 | French Giana | Testis | Illumina HiSeq2000 |
|  | *Caecilia tentaculata* | Amphibian | SRR5591446 | French Giana | Spleen | Illumina HiSeq2000 |
|  | *Caecilia tentaculata* | Amphibian | SRR5591447 | French Giana | Muscle | Illumina HiSeq2000 |
|  | *Caecilia tentaculata* | Amphibian | SRR5591448 | French Giana | Lung | Illumina HiSeq2000 |
|  | *Caecilia tentaculata* | Amphibian | SRR5591449 | French Giana | Skin | Illumina HiSeq2000 |
|  | *Caecilia tentaculata* | Amphibian | SRR5591450 | French Giana | Skin | Illumina HiSeq2000 |
|  | *Caecilia tentaculata* | Amphibian | SRR5591451 | French Giana | Heart | Illumina HiSeq2000 |
|  | *Caecilia tentaculata* | Amphibian | SRR5591452 | French Giana | Foregut | Illumina HiSeq2000 |
|  | *Caecilia tentaculata* | Amphibian | SRR5591453 | French Giana | Liver | Illumina HiSeq2000 |
|  | *Caecilia tentaculata* | Amphibian | SRR5591454 | French Giana | Kidney | Illumina HiSeq2000 |
| Tailed frog | *Ascaphus truei* | Amphibian | SRR9590994 | USA | Testes | Illumina HiSeq4000 |
| Oriental fire-bellied toad | *Bombina orientalis* | Amphibian | ERR632225 | UK | Liver | Illumina HiSeq 2500 |
| Yellow bellied toad | *Bombina variegata scabra* | Amphibian | ERR632224 | UK | Liver | Illumina HiSeq 2500 |
| Fire-bellied toad | *Bombina bombina* | Amphibian | ERR632222 | UK | Liver | Illumina HiSeq 2500 |
| Sikkim lazy toad | *Scutiger* | Amphibian | SRR9953600 | Nepal | Testes | NextSeq 500 |
| Eastern spadefoot toad | *Leptobrachium ailaonicum* | Amphibian | SRR8720654 | China | Mixed viscera | Illumina HiSeq 4000 |
| Omei lazy toad | *Oreolalax omeimontis* | Amphibian | SRR8991298 | China | Mixed tadpole viscera | Illumina HiSeq 2500 |
| Common lazy toad | *Oreolalax major* | Amphibian | SRR8991299 | China | Mixed tadpole viscera | Illumina HiSeq 2500 |
| Guizhou lazy toad | *Oreolalax rhodostigmatus* | Amphibian | SRR8991300 | China | Mixed tadpole viscera | Illumina HiSeq 2500 |
| Pope's lazy toad | *Oreolalax popei* | Amphibian | SRR8991301 | China | Mixed tadpole viscera | Illumina HiSeq 2500 |
| Omei horned toad | *Megophrys omeimontis* | Amphibian | SRR8991302 | China | Mixed tadpole viscera | Illumina HiSeq 2500 |
| Plains spadefoot toad | *Spea bombifrons* | Amphibian | SRR9160212 | USA | Tadpole | NextSeq 500 |
| New Mexico spadefoot toad | *Spea multiplicata* | Amphibian | SRR9160218 | USA | Tadpole | NextSeq 500 |
| Bicolored dart frog | *Phyllobates bicolor* | Amphibian | SRR12232938 | Colombia | Mixed viscera | Illumina HiSeq 2500 |
| Sira poison frog | *Ranitomeya sirensis* | Amphibian | ERR4074563 | Peru | Liver | NextSeq 500 |
| Chinese giant salamander | *Andrias davidianus* | Amphibian | SRR5944939 | China | Lung | HiSeq X Ten |
| Tuatara | *Sphenodon punctatus* | Reptile | SRR7084910 | New Zealand | Blood | Illumina HiSeq 2500 |
| Madagascar ground gecko | *Paroedura picta* | Reptile | DRR047251 | Madagascar | Embryo | Illumina HiSeq 1500 |
| Bearded pygmy chameleon | *Rieppeleon brevicaudatus* | Reptile | SRR9298913 | Madagascar | Mixed viscera | NextSeq 500 |
| Perinet chameleon | *Calumma gastrotaenia* | Reptile | SRR9298915 | Madagascar | Mixed viscera | NextSeq 500 |
| Bohme's two-horned chameleon | *Kinyongia boehmei* | Reptile | SRR9298917 | Madagascar | Mixed viscera | NextSeq 500 |
| Nguru pygmy chameleon | *Rhampholeon acuminatus* | Reptile | SRR9298918 | Madagascar | Mixed viscera | NextSeq 500 |
| von Hohnel's chameleon | *Trioceros hoehnelii* | Reptile | SRR9298919 | Madagascar | Mixed viscera | NextSeq 500 |
| Brown leaf chameleon | *Brookesia superciliaris* | Reptile | SRR9298920 | Madagascar | Mixed viscera | NextSeq 500 |
| Cape dwarf chameleon | *Bradypodion pumilum* | Reptile | SRR9298921 | Madagascar | Mixed viscera | NextSeq 500 |
|  | *Calumma ambreense* | Reptile | SRR9298922 | Madagascar | Mixed viscera | NextSeq 500 |
| Panther chameleon | *Furcifer pardalis* | Reptile | SRR9298914 | Madagascar | Mixed viscera | NextSeq 500 |
| Brown basilisk | *Basiliscus vittatus* | Reptile | SRR8700057 | Mexico | Liver | Illumina HiSeq 2000* |
| Iguana | *Iguana iguana* | Reptile | SRR1693196 | Unknown | Blood | Illumina HiSeq 2000* |
| Chinese alligator | *Alligator sinensis* | Reptile | SRR4212883 | China | Kidney | Illumina HiSeq 4000 |
| Spectacled caiman | *Caiman crorodilus* | Reptile | ERR2198478 | Unknown | Liver | Illumina HiSeq 2000 |
| Cape coral snake | *Aspidelaps lubricus cowlesi* | Reptile | SRR8877650 | Netherlands | Liver | Illumina HiSeq 2500* |
| Central Asian pit viper | *Gloydius intermedius* | Reptile | SRR8272683 | China | Mixed viscera | Illumina HiSeq 2000 |
| Shedao island pit viper | *Gloydius shedaoensis* | Reptile | SRR8272684 | China | Mixed viscera | Illumina HiSeq 2000 |
| Boa contstrictor | *Boa constrictor* | Reptile | SRR1693194 | Unknown | Blood | Illumina HiSeq 2000 |
| Indian python | *Python molurus* | Reptile | SRR11149665 | Unknown | Testis | Illumina HiSeq 2500 |
| Coorg night frog | *Nyctibatrachus sanctipalustris* | Amphibian | SRR8954550 | India | Nuptial pad | Illumina HiSeq 2500 |
| Common toad | *Bufo bufo* | Amphibian | SRR8954551 | Unknown | Nuptial pad | Illumina HiSeq 2500 |
| Accra snake-necked frog | *Phrynomantis microps* | Amphibian | SRR8954552 | Africa | Dorsal skin | Illumina HiSeq 2500 |
| Demerara falls tree frog | *Boana cinerascens* | Amphibian | SRR8954553 | Peru | Mental gland | Illumina HiSeq 2500 |
| Edible frog | *Pelophylax esculentus* | Amphibian | SRR8954554 | France | Nuptial pad | Illumina HiSeq 2500 |
| Clown tree frog | *Dendropsophus sarayacuensis* | Amphibian | SRR8954555 | Peru | Pectoral gland | Illumina HiSeq 2500 |
| Congo dwarf clawed frog | *Hymenochirus boettgeri* | Amphibian | SRR8954556 | Africa | Axillary gland | Illumina HiSeq 2500 |
| Roque tree frog | *Hyloscirtus phyllognathus* | Amphibian | SRR8954557 | Peru | Mental gland | Illumina HiSeq 2500 |
| Wayanad night frog | *Nyctibatrachus grandis* | Amphibian | SRR8954558 | India | Nuptial pad | Illumina HiSeq 2500 |
| Mallans dancing frog | *Micrixalus mallani* | Amphibian | SRR8954559 | India | Nuptial pad | Illumina HiSeq 2500 |
| Castle rock night frog | *Nyctibatrachus petraeus* | Amphibian | SRR8954560 | India | Nuptial pad | Illumina HiSeq 2500 |
| Castle rock night frog | *Nyctibatrachus petraeus* | Amphibian | SRR8954561 | India | Femoral gland | Illumina HiSeq 2500 |
| Oriental fire-bellied toad | *Bombina orientalis* | Amphibian | SRR8954562 | India | Nuptial pad | Illumina HiSeq 2500 |
| Coorg night frog | *Nyctibatrachus sanctipalustris* | Amphibian | SRR8954563 | India | Femoral gland | Illumina HiSeq 2500 |
| Amboli bush frog | *Pseudophilautus amboli* | Amphibian | SRR8954564 | India | Ventral skin | Illumina HiSeq 2500 |
|  | *Fejervarya goemchi* | Amphibian | SRR8954565 | India | Nuptial pad | Illumina HiSeq 2500 |
| Amboli leaping frog | *Indirana chiravasi* | Amphibian | SRR8954566 | India | Nuptial pad | Illumina HiSeq 2500 |
| Amboli leaping frog | *Indirana chiravasi* | Amphibian | SRR8954567 | India | Femoral gland | Illumina HiSeq 2500 |
| Tuatara | *Sphenodon punctatus* | Reptile | SRR485948 | New Zealand | Embryo | Illumina Genome Analyzer IIx |
| Brandt's Persian lizard | *Iranolacerta brandtii* | Reptile | SRR9090238 | Iran | Mixed viscera | NextSeq 500 |
| Sand lizard | *Lacerta agilis* | Reptile | SRR9090239 | Europe | Mixed viscera | NextSeq 500 |
| North African ocellated lizard | *Timon pater* | Reptile | SRR9090240 | Africa | Mixed viscera | NextSeq 500 |
| Asian grass lizard | *Takydromus sexlineatus* | Reptile | SRR9090241 | Asia | Tail fin | NextSeq 500 |
| Red-bellied lizard | *Darevskia parvula* | Reptile | SRR9090242 | Turkey | Mixed viscera | NextSeq 500 |
| Sawtail lizard | *Holaspis guentheri* | Reptile | SRR9090243 | Africa | Mixed viscera | NextSeq 500 |
| European worm lizard | *Blanus cinereus* | Reptile | SRR9090244 | Spain | Mixed viscera | NextSeq 500 |
| Pyrenean rock lizard | *Iberolacerta bonnali* | Reptile | SRR9090245 | Spain | Mixed viscera | NextSeq 500 |
| Algerian psammodromus | *Psammodromus algirus* | Reptile | SRR9090246 | Spain | Mixed viscera | NextSeq 500 |
| Catalan wall lizard | *Podarcis liolepis* | Reptile | SRR9090247 | Spain | Mixed viscera | NextSeq 500 |
| Common wall lizard | *Podarcis muralis* | Reptile | SRR9090248 | Europe | Mixed viscera | NextSeq 500 |
| Anatolian lizard | *Apathya cappadocica* | Reptile | SRR9090249 | Turkey | Mixed viscera | NextSeq 500 |
| Lebanon lizard | *Phoenicolacerta laevis* | Reptile | SRR9090250 | Turkey | Mixed viscera | NextSeq 500 |
| Sharp-snouted rock lizard | *Dalmatolacerta oxycephala* | Reptile | SRR9090251 | Croatia | Mixed viscera | NextSeq 500 |
| Mosor rock lizard | *Dinarolacerta mosorensis* | Reptile | SRR9090252 | Croatia | Mixed viscera | NextSeq 500 |
| Bedriaga's rock lizard | *Archaeolacerta bedriagae* | Reptile | SRR9090253 | Italy | Mixed viscera | NextSeq 500 |
| Blue-throated keeled lizard | *Algyroides nigropunctatus* | Reptile | SRR9090254 | Croatia | Mixed viscera | NextSeq 500 |
| Greek rock lizard | *Hellenolacerta graeca* | Reptile | SRR9090255 | Greece | Mixed viscera | NextSeq 500 |
| Viviparous lizard | *Zootoca vivipara* | Reptile | SRR9090256 | France | Mixed viscera | NextSeq 500 |
| Moroccan rock lizard | *Scelarcis perspicillata* | Reptile | SRR9090257 | Morocco | Mixed viscera | NextSeq 500 |
| Gallot's lizard | *Gallotia galloti* | Reptile | SRR9090258 | Spain | Mixed viscera | NextSeq 500 |
| Copperhead pit viper | *Agkistrodon contortrix* | Reptile | SRR188168 | USA | Liver | 454 GS FLX* |
| Cottonmouth snake | *Agkistrodon piscivorus* | Reptile | SRR629645 | USA | Liver | Illumina HiSeq 2000* |
| American alligator | *Alligator mississippiensis* | Reptile | SRR629636 | USA | Liver | Illumina HiSeq 2000* |
| Chinese alligator | *Alligator sinensis* | Reptile | SRR957515 | China | Blood | Illumina Genome Analyzer II |
| Chinese alligator | *Alligator sinensis* | Reptile | SRR4210197 | China | Kidney | Illumina HiSeq 4000 |
| Chinese alligator | *Alligator sinensis* | Reptile | SRR4212880 | China | Kidney | Illumina HiSeq 4000 |
| Chinese alligator | *Alligator sinensis* | Reptile | SRR4212883 | China | Kidney | Illumina HiSeq 4000 |
| Bueycito anole | *Anolis allogus* | Reptile | DRR055059 | Cuba | Liver | Illumina HiSeq 2000 |
| Bueycito anole | *Anolis allogus* | Reptile | DRR055060 | Cuba | Liver | Illumina HiSeq 2000 |
| Bueycito anole | *Anolis allogus* | Reptile | DRR055061 | Cuba | Liver | Illumina HiSeq 2000 |
| Bueycito anole | *Anolis allogus* | Reptile | DRR055063 | Cuba | Liver | Illumina HiSeq 2000 |
| Bueycito anole | *Anolis allogus* | Reptile | DRR055062 | Cuba | Liver | Illumina HiSeq 2000 |
| Bueycito anole | *Anolis allogus* | Reptile | DRR055064 | Cuba | Liver | Illumina HiSeq 2000 |
| Bueycito anole | *Anolis allogus* | Reptile | DRR055065 | Cuba | Liver | Illumina HiSeq 2000 |
| Bueycito anole | *Anolis allogus* | Reptile | DRR055066 | Cuba | Liver | Illumina HiSeq 2000 |
| Cuban White-fanned Anole | *Anolis homolechis* | Reptile | DRR055083 | Cuba | Liver | Illumina HiSeq 2000 |
| Cuban White-fanned Anole | *Anolis homolechis* | Reptile | DRR055085 | Cuba | Liver | Illumina HiSeq 2000 |
| Cuban White-fanned Anole | *Anolis homolechis* | Reptile | DRR055086 | Cuba | Liver | Illumina HiSeq 2000 |
| Cuban White-fanned Anole | *Anolis homolechis* | Reptile | DRR055087 | Cuba | Liver | Illumina HiSeq 2000 |
| Cuban White-fanned Anole | *Anolis homolechis* | Reptile | DRR055084 | Cuba | Liver | Illumina HiSeq 2000 |
| Cuban White-fanned Anole | *Anolis homolechis* | Reptile | DRR055088 | Cuba | Liver | Illumina HiSeq 2000 |
| Cuban White-fanned Anole | *Anolis homolechis* | Reptile | DRR055090 | Cuba | Liver | Illumina HiSeq 2000 |
| Cuban White-fanned Anole | *Anolis homolechis* | Reptile | DRR055089 | Cuba | Liver | Illumina HiSeq 2000 |
| Brown anole | *Anolis sagrei* | Reptile | SRR629522 | Unknown | Liver | Illumina HiSeq 2000* |
| Spiny softshell turtle | *Apalone spinifera* | Reptile | SRR5242255 | USA | Kidney | Illumina HiSeq 2000 |
| Spiny softshell turtle | *Apalone spinifera* | Reptile | SRR5242256 | USA | Kidney | Illumina HiSeq 2000 |
| Spiny softshell turtle | *Apalone spinifera* | Reptile | SRR5242260 | USA | Kidney | Illumina HiSeq 2000 |
| Spiny softshell turtle | *Apalone spinifera* | Reptile | SRR5242261 | USA | Kidney | Illumina HiSeq 2000 |
| Pit viper | *Bothrops jararaca* | Reptile | SRR1596200 | Brazil | Kidney | 454 GS Junior |
| Pit viper | *Bothrops jararaca* | Reptile | SRR1596064 | Brazil | Liver | 454 GS Junior |
| Pit viper | *Bothrops jararaca* | Reptile | SRR1596065 | Brazil | Pancreas | 454 GS Junior |
| Pit viper | *Bothrops jararaca* | Reptile | SRR1596066 | Brazil | Pancreas | 454 GS Junior |
| Viper boa | *Candoia aspera* | Reptile | SRR629646 | Unknown | Liver | Illumina HiSeq 2000* |
| Loggerhead sea turtle | *Caretta caretta* | Reptile | SRR5330501 | Colombia | Blood | Illumina HiSeq 2000* |
| Common snapping turtle | *Cheyldra serpentina* | Reptile | SRR629521 | Unknown | Liver | Illumina HiSeq 2000* |
| Spiny softshell turtle | *Chrysemys picta* | Reptile | SRR5242265 | USA | Kidney | Illumina HiSeq 2000 |
| Spiny softshell turtle | *Chrysemys picta* | Reptile | SRR5242266 | USA | Kidney | Illumina HiSeq 2000 |
| Spiny softshell turtle | *Chrysemys picta* | Reptile | SRR5242270 | USA | Kidney | Illumina HiSeq 2000 |
| Spiny softshell turtle | *Chrysemys picta* | Reptile | SRR5242271 | USA | Kidney | Illumina HiSeq 2000 |
| Siamese crocodile | *Crocodylus siamensis* | Reptile | SRR1612398 | China | Mixed viscera | Illumina HiSeq 2000 |
| Chinese crocodile | *Crocodylus siamensis* | Reptile | SRR1693772 | China | Mixed viscera | Illumina HiSeq 2000 |
| Southern Alligator lizard | *Elgaria multicarinata* | Reptile | SRR629637 | Unknown | Liver | Illumina HiSeq 2000* |
| Hawksbill sea turtle | *Eretmochelys imbricata* | Reptile | SRR5357800 | Colombia | Blood | Illumina HiSeq 2000* |
| Leopard gecko | *Eublepharis macularius* | Reptile | SRR629643 | Unknown | Liver | Illumina HiSeq 2000* |
| Green iguana | *Gambelia wislizenii* | Reptile | SRR7830697 | Mexico | Kidney | Illumina HiSeq 2000* |
| Green iguana | *Iguana iguana* | Reptile | SRR7830704 | Mexico | Kidney | Illumina HiSeq 2000* |
| Green iguana | *Gambelia wislizenii* | Reptile | SRR7830700 | Mexico | Liver | Illumina HiSeq 2000* |
| Green iguana | *Iguana iguana* | Reptile | SRR7830703 | Mexico | Liver | Illumina HiSeq 2000* |
| African House Snake | *Lamprophis sp.* | Reptile | SRR629644 | Unknown | Liver | Illumina HiSeq 2000 |
| Chinese pond turtle | *Mauremys reevesii* | Reptile | SRR955754 | China | Mixed viscera | Illumina HiSeq 2000 |
| Chinese pond turtle | *Mauremys reevesii* | Reptile | SRR2980465 | China | Mixed viscera | Illumina HiSeq 2500 |
| Tiger snake | *Notechis scutatus* | Reptile | SRR519122 | Australia | Liver | Illumina HiSeq 2000 |
| Tiger snake | *Notechis scutatus* | Reptile | SRR519464 | Australia | Liver | Illumina HiSeq 2000 |
| Chinese softshell turtle | *Pelodiscus sinensis* | Reptile | SRR6157006 | China | Liver | Illumina HiSeq 4000* |
| Chinese softshell turtle | *Pelodiscus sinensis* | Reptile | SRR6180859 | China | Liver | Illumina HiSeq 4000* |
| Chinese softshell turtle | *Pelodiscus sinensis* | Reptile | SRR6180860 | China | Liver | Illumina HiSeq 4000* |
| Chinese softshell turtle | *Pelodiscus sinensis* | Reptile | SRR6180861 | China | Liver | Illumina HiSeq 4000* |
| Chinese softshell turtle | *Pelodiscus sinensis* | Reptile | SRR6180862 | China | Liver | Illumina HiSeq 4000* |
| Chinese softshell turtle | *Pelodiscus sinensis* | Reptile | SRR6180863 | China | Liver | Illumina HiSeq 4000* |
| Chinese softshell turtle | *Pelodiscus sinensis* | Reptile | SRR6180864 | China | Liver | Illumina HiSeq 4000* |
| Chinese softshell turtle | *Pelodiscus sinensis* | Reptile | SRR6180865 | China | Liver | Illumina HiSeq 4000* |
| Chinese softshell turtle | *Pelodiscus sinensis* | Reptile | SRR6180866 | China | Liver | Illumina HiSeq 4000* |
| Chinese softshell turtle | *Pelodiscus sinensis* | Reptile | SRR6180867 | China | Liver | Illumina HiSeq 4000* |
| Chinese softshell turtle | *Pelodiscus sinensis* | Reptile | SRR6180868 | China | Liver | Illumina HiSeq 4000* |
| Sideneck turtle | *Pelusios castaneus* | Reptile | SRR629649 | Unknown | Liver | Illumina HiSeq 2000* |
| Bearded dragon | *Pogona vitticeps* | Reptile | SRR629641 | Unknown | Liver | Illumina HiSeq 2000* |
| Burmese python | *Python bivittatus* | Reptile | SRR5434342 | USA | Small intestine | Illumina HiSeq 2500* |
| Burmese python | *Python bivittatus* | Reptile | SRR4280477 | Denmark | Small intestine | Illumina HiSeq 2000 |
| Burmese python | *Python bivittatus* | Reptile | SRR4280482 | Denmark | Small intestine | Illumina HiSeq 2000 |
| Burmese python | *Python bivittatus* | Reptile | SRR4280488 | Denmark | Small intestine | Illumina HiSeq 2000 |
| Burmese python | *Python bivittatus* | Reptile | SRR4280490 | Denmark | Small intestine | Illumina HiSeq 2000 |
| Burmese python | *Python bivittatus* | Reptile | SRR4280495 | Denmark | Small intestine | Illumina HiSeq 2000 |
| Burmese python | *Python bivittatus* | Reptile | SRR4280473 | Denmark | Liver | Illumina HiSeq 2000 |
| Burmese python | *Python bivittatus* | Reptile | SRR4280475 | Denmark | Liver | Illumina HiSeq 2000 |
| Burmese python | *Python bivittatus* | Reptile | SRR4280480 | Denmark | Liver | Illumina HiSeq 2000 |
| Burmese python | *Python bivittatus* | Reptile | SRR4280486 | Denmark | Liver | Illumina HiSeq 2000 |
| Burmese python | *Python bivittatus* | Reptile | SRR4280493 | Denmark | Liver | Illumina HiSeq 2000 |
| Indian python | *Python molurus* | Reptile | SRR188171 | Unknown | Liver | 454 GS FLX* |
| Fence lizard | *Sceloporus undulatus* | Reptile | SRR629640 | Unknown | Liver | Illumina HiSeq 2000* |
| Skink | *Scincella lateralis* | Reptile | SRR629642 | Unknown | Liver | Illumina HiSeq 2000* |
| Sunbeam snake | *Xenopeltis unicolor* | Reptile | SRR629647 | Unknown | Liver | Illumina HiSeq 2000* |
| Stinkpot turtle | *Sternotherus odoratus* | Reptile | SRR629648 | Unknown | Liver | Illumina HiSeq 2000* |
| Eastern box turtle | *Terrapene carolina* | Reptile | SRR629650 | Unknown | Liver | Illumina HiSeq 2000* |
| Sierra garter snake | *Thamnophis couchii* | Reptile | SRR629634 | Unknown | Liver | Illumina HiSeq 2000* |
| Western terrestrial garter snake | *Thamnophis elegans* | Reptile | SRR497737 | USA | Liver | Illumina Genome Analyzer II |
| Western terrestrial garter snake | *Thamnophis elegans* | Reptile | SRR497738 | USA | Liver | Illumina Genome Analyzer II |
| Western terrestrial garter snake | *Thamnophis elegans* | Reptile | SRR497739 | USA | Liver | Illumina Genome Analyzer II |
| Western terrestrial garter snake | *Thamnophis elegans* | Reptile | SRR497740 | USA | Liver | Illumina Genome Analyzer II |
| Western terrestrial garter snake | *Thamnophis elegans* | Reptile | SRR497741 | USA | Liver | Illumina Genome Analyzer II |
| Western terrestrial garter snake | *Thamnophis elegans* | Reptile | SRR497742 | USA | Liver | Illumina Genome Analyzer II |
| Western terrestrial garter snake | *Thamnophis elegans* | Reptile | SRR497743 | USA | Liver | Illumina Genome Analyzer II |
| Western terrestrial garter snake | *Thamnophis elegans* | Reptile | SRR497744 | USA | Liver | Illumina Genome Analyzer II |
| Western terrestrial garter snake | *Thamnophis elegans* | Reptile | SRR497745 | USA | Liver | Illumina Genome Analyzer II |
| Western terrestrial garter snake | *Thamnophis elegans* | Reptile | SRR497746 | USA | Liver | Illumina Genome Analyzer II |
| Western terrestrial garter snake | *Thamnophis elegans* | Reptile | SRR497747 | USA | Liver | Illumina Genome Analyzer II |
| Western terrestrial garter snake | *Thamnophis elegans* | Reptile | SRR497748 | USA | Liver | Illumina Genome Analyzer II |
| Western terrestrial garter snake | *Thamnophis elegans* | Reptile | SRR497749 | USA | Liver | Illumina Genome Analyzer II |
| Red-eared slider | *Trachemys scripta* | Reptile | ERR2198830 | Denmark | Liver | Illumina HiSeq 2000 |
| Red-eared slider | *Trachemys scripta elegans* | Reptile | SRR7540569 | China | Liver | Illumina HiSeq 4000 |
| Red-eared slider | *Trachemys scripta elegans* | Reptile | SRR7540570 | China | Liver | Illumina HiSeq 4000 |
| Red-eared slider | *Trachemys scripta elegans* | Reptile | SRR7540571 | China | Liver | Illumina HiSeq 4000 |
| Japanese fire belly newt | *Cynops pyrrhogaster* | Amphibian | SRR1553357 | Japan | Mixed viscera | Illumina HiSeq 2500* |
| Komodo dragon | *Varanus komodensis* | Reptile | SRR8735151 | Unknown | Heart | NextSeq 500 |
| Komodo dragon | *Varanus komodensis* | Reptile | SRR8735152 | Unknown | Heart | NextSeq 500 |
| Central bearded dragon | *Pogona vitticeps* | Reptile | SRR8925842 | Australia | Heart | NextSeq 500 |
| Central bearded dragon | *Pogona vitticeps* | Reptile | SRR8925843 | Australia | Heart | NextSeq 500 |
| Central bearded dragon | *Pogona vitticeps* | Reptile | SRR8925844 | Australia | Heart | NextSeq 500 |
| Central bearded dragon | *Pogona vitticeps* | Reptile | SRR8925845 | Australia | Heart | NextSeq 500 |
| Tawny snake eyed skink | *Crytoblepharus ruber* | Reptile | SRR3901727 | Australia | Liver | Illumina HiSeq 2000 |
| Eastern striped skink | *Ctenotus robustus* | Reptile | SRR3901725 | Australia | Liver | Illumina HiSeq 2000 |
| Broad-banded sand-swimmer | *Eremiascincus ciliaris* | Reptile | SRR3901726 | Australia | Liver | Illumina HiSeq 2000 |
| Northern spotted rock dtella | *Gehyra nana* | Reptile | SRR3901730 | Australia | Liver | Illumina HiSeq 2000 |
| Marbled velvet gecko | *Oedura marmorata* | Reptile | SRR3901728 | Australia | Liver | Illumina HiSeq 2000 |
| Northern spiny-tailed gecko | *Strophurus ciliaris* | Reptile | SRR3901729 | Australia | Liver | Illumina HiSeq 2000 |
| Spiny-tailed monitor | *Varanas acanthurus* | Reptile | SRR3901711 | Australia | Liver | Illumina HiSeq 2000 |
| Desert tree frog | *Litoria rubella* | Amphibian | SRR3901722 | Australia | Liver | Illumina HiSeq 2000 |
| Robust frog | *Austrochaperina robusta* | Amphibian | SRR3901723 | Australia | Liver | Illumina HiSeq 2000 |
| Giant frog | *Cyclorana australis* | Amphibian | SRR3901720 | Australia | Liver | Illumina HiSeq 2000 |
| Mahony's toadlet | *Uperoleia mahonyi* | Amphibian | SRR3901719 | Australia | Liver | Illumina HiSeq 2000 |
| * indicates PolyA selection was used during library preparation |  |  |  |  |  |  |


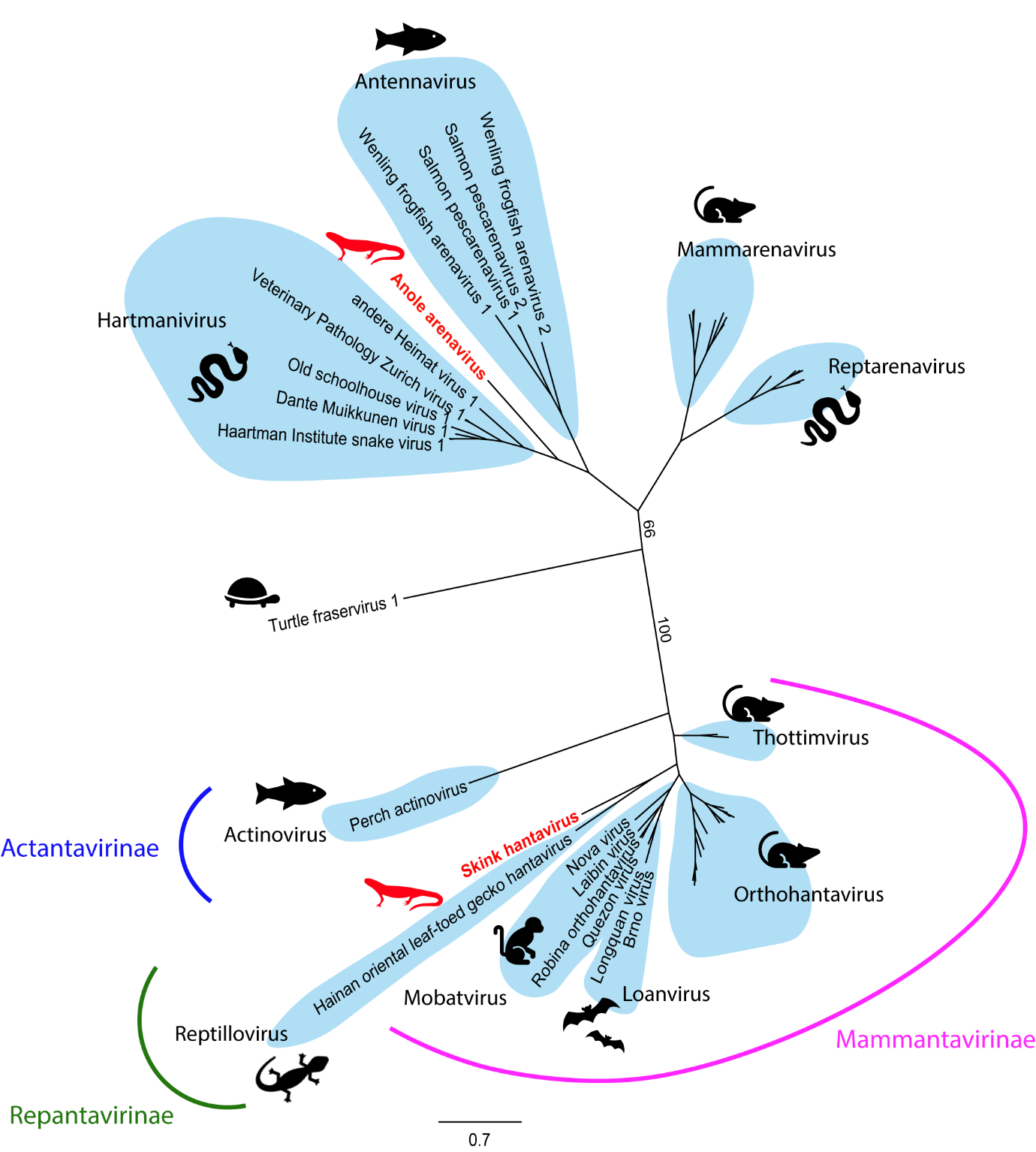


***Supplementary Figure 1: Novel reptile* Bunyavirales*.*** *Contigs were identified using a BLAST search of annotated bunyavirales proteins downloaded from NCBI protein database against herptile transcriptomes* ***B:*** *The nucleoprotein gene of bunyavirales were* in silico *translated and 582 AA was aligned with reference viruses using MAFFT. Phylogenetic trees were constructed using RAxML with 500 bootstrap replicates. Novel viruses are red and known classifications are shaded. The scale bar represents substitutions per site.*


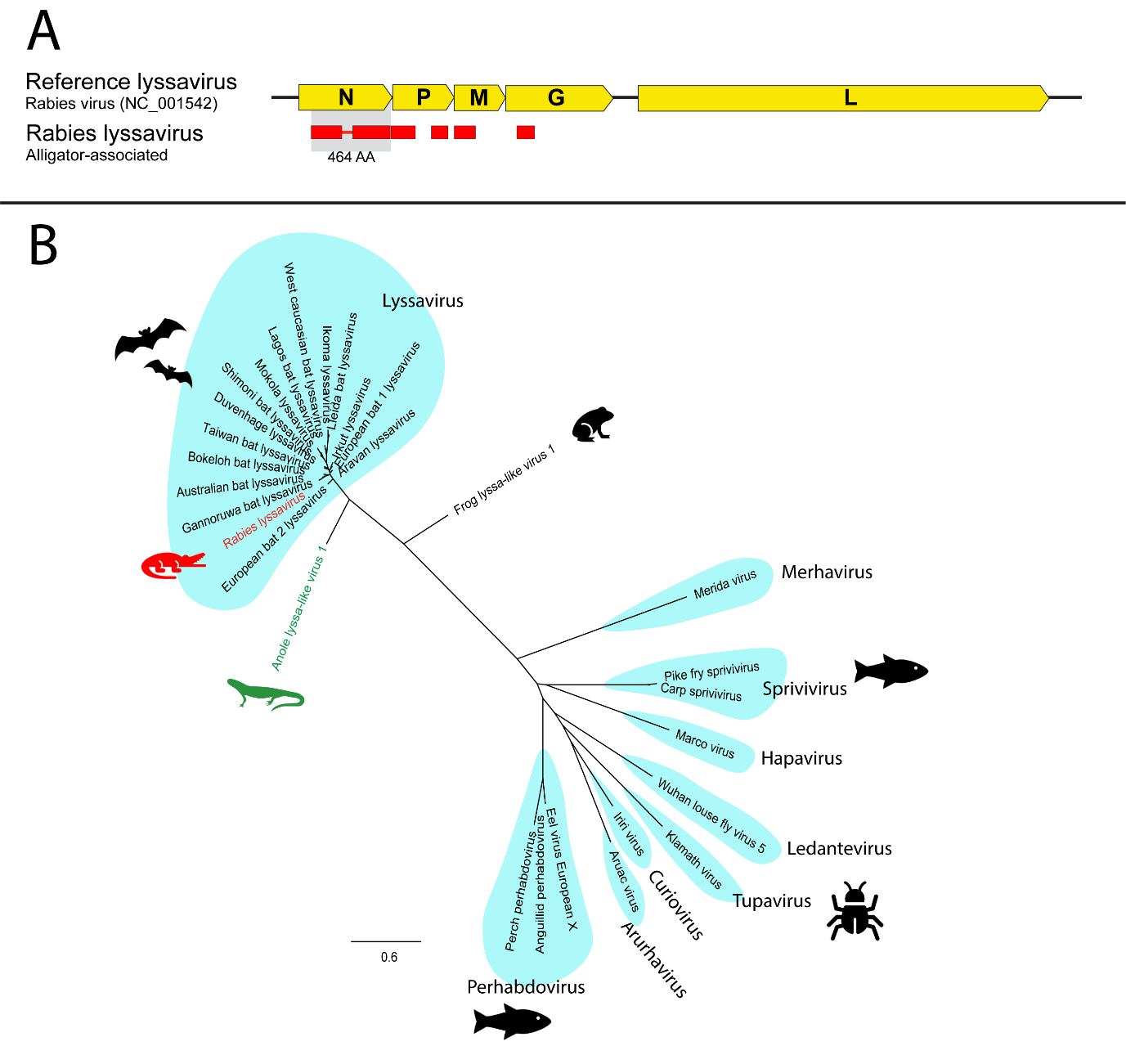


***Supplementary Figure 2: Reptile lyssaviruses.*** *Contigs were identified using a BLAST search of rhabdovirus proteins downloaded from NCBI protein database against transcriptomes.* ***A: Genome of Rabies lyssavirus.*** *Viral contigs (red) were aligned with the reference genome (yellow) using MAFFT. The grey box represents the region selected for phylogenetic analysis.* ***B: Phylogeny of rhabdoviruses.*** *The nucleoprotein (N) gene of anole lyssa-like virus and alligator-associated rabies lyssavirus was* in silico *translated and aligned with reference rhabdoviruses using MAFFT. A phylogenetic tree was constructed using RAxML with 500 bootstrap replicates. Alligator-associated rabies lyssavirus is coloured red and the previously identified anole lyssa-like virus is green. Known classifications are shaded. The scale bar represents substitutions per site.*


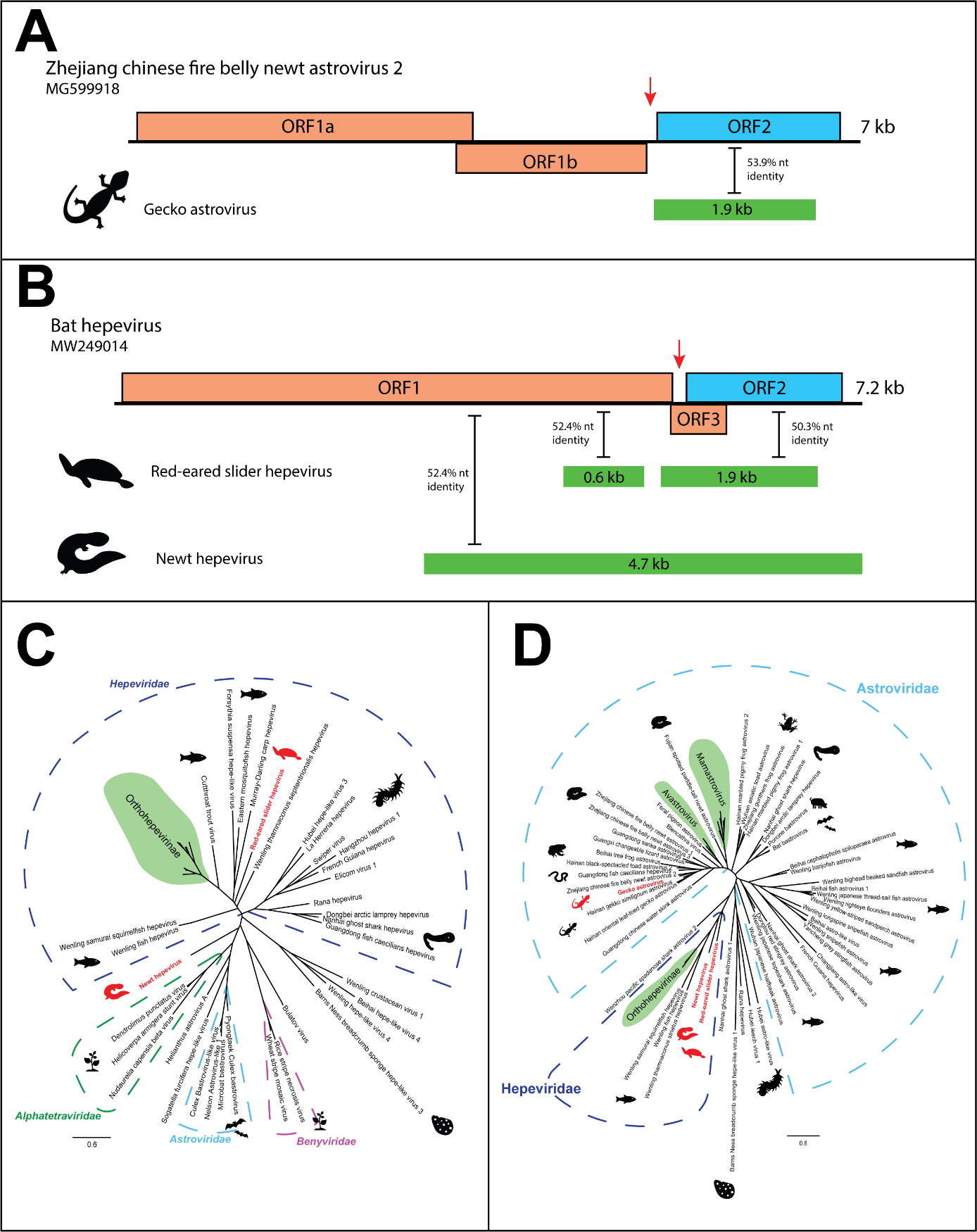


***Supplementary Figure 3: Novel amphibian and reptile* Hepeviridae *and* Astroviridae.** *Contigs were identified using a BLAST search of astrovirus and hepevirus proteins downloaded from NCBI protein database against herptile transcriptomes.* ***A: Astrovirus genomic structure.*** *Virus contigs (green) were aligned with reference genomes using MAFFT. Non-structural genes are shaded orange and structural genes are shaded blue. The common recombination breakpoint is denoted with a red arrow.* ***B: Hepevirus genomic structure.*** *Virus contigs (green) were aligned with reference genomes using MAFFT. Non-structural genes are shaded orange and structural genes are shaded blue. The common recombination breakpoint is denoted with a red arrow.* ***C: Non-structural gene*** ***phylogeny of astroviruses and hepeviruses.*** *The non-structural gene of novel viruses were in silico translated and aligned with reference viruses using MAFFT. A phylogenetic tree was constructed using RAxML with 500 bootstrap replicates. Novel viruses are coloured red and known classifications are shaded or outlined. The scale bar represents substitutions per site.* ***D: Structural phylogeny of hepeviruses.*** *The structural capsid genes of novel viruses were in silico translated and aligned with reference viruses using MAFFT. A phylogenetic tree was constructed using RAxML with 500 bootstrap replicates. Novel viruses are coloured red and known classifications are shaded and outlined. The scale bar represents substitutions per site.*

*
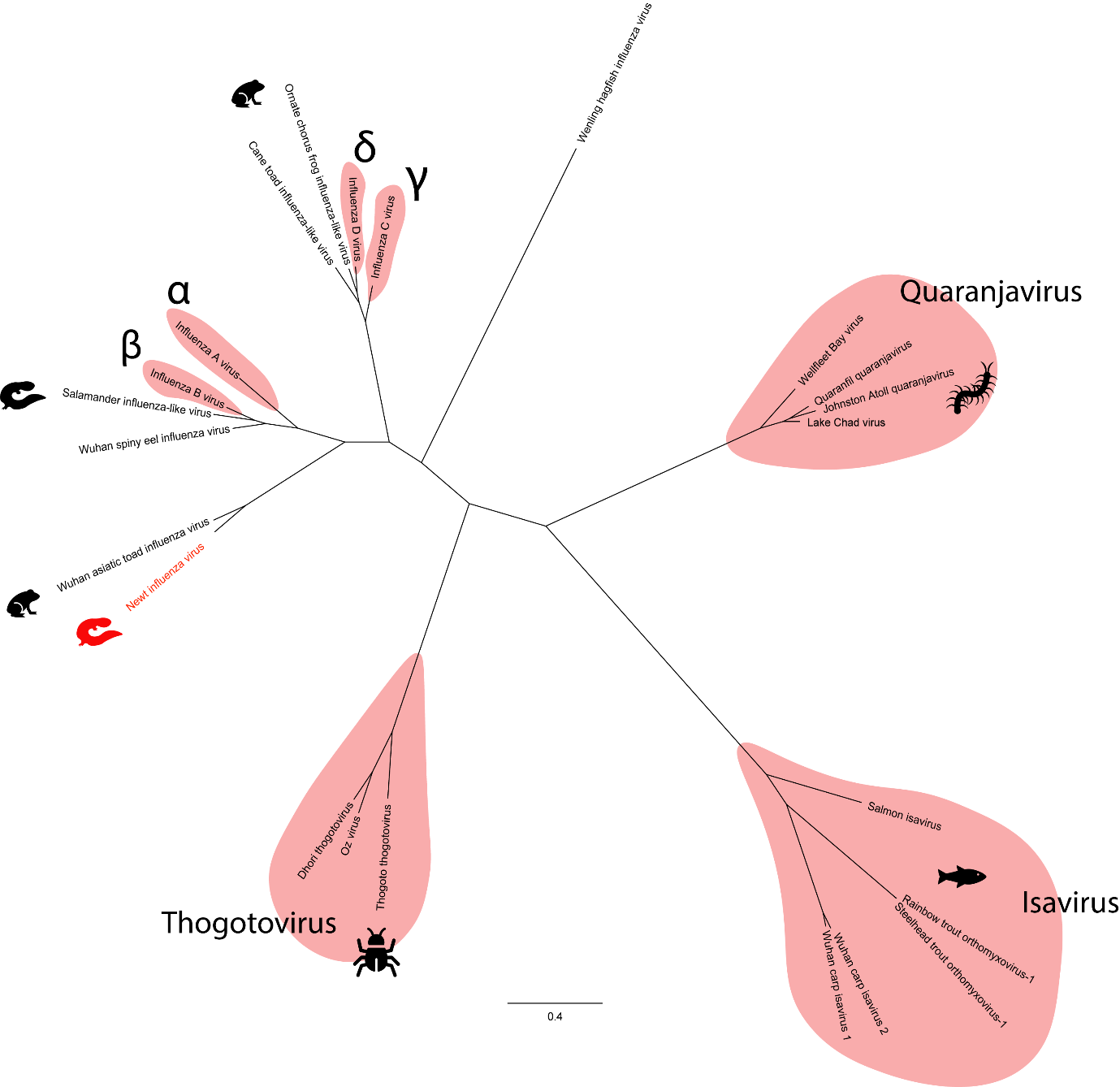
*

***Supplementary Figure 4: Novel newt influenza virus.*** *Contigs were identified using a BLAST search of influenza virus proteins downloaded from NCBI protein database against herptile transcriptomes. The polymerase basic 1 (PB1) gene of newt influenza virus was in silico translated and aligned with reference orthomyxoviruses using MAFFT. A phylogenetic tree was constructed using RAxML with 500 bootstrap replicates. Newt influenza virus is coloured red and known classifications are shaded. The scale bar represents substitutions per site.*


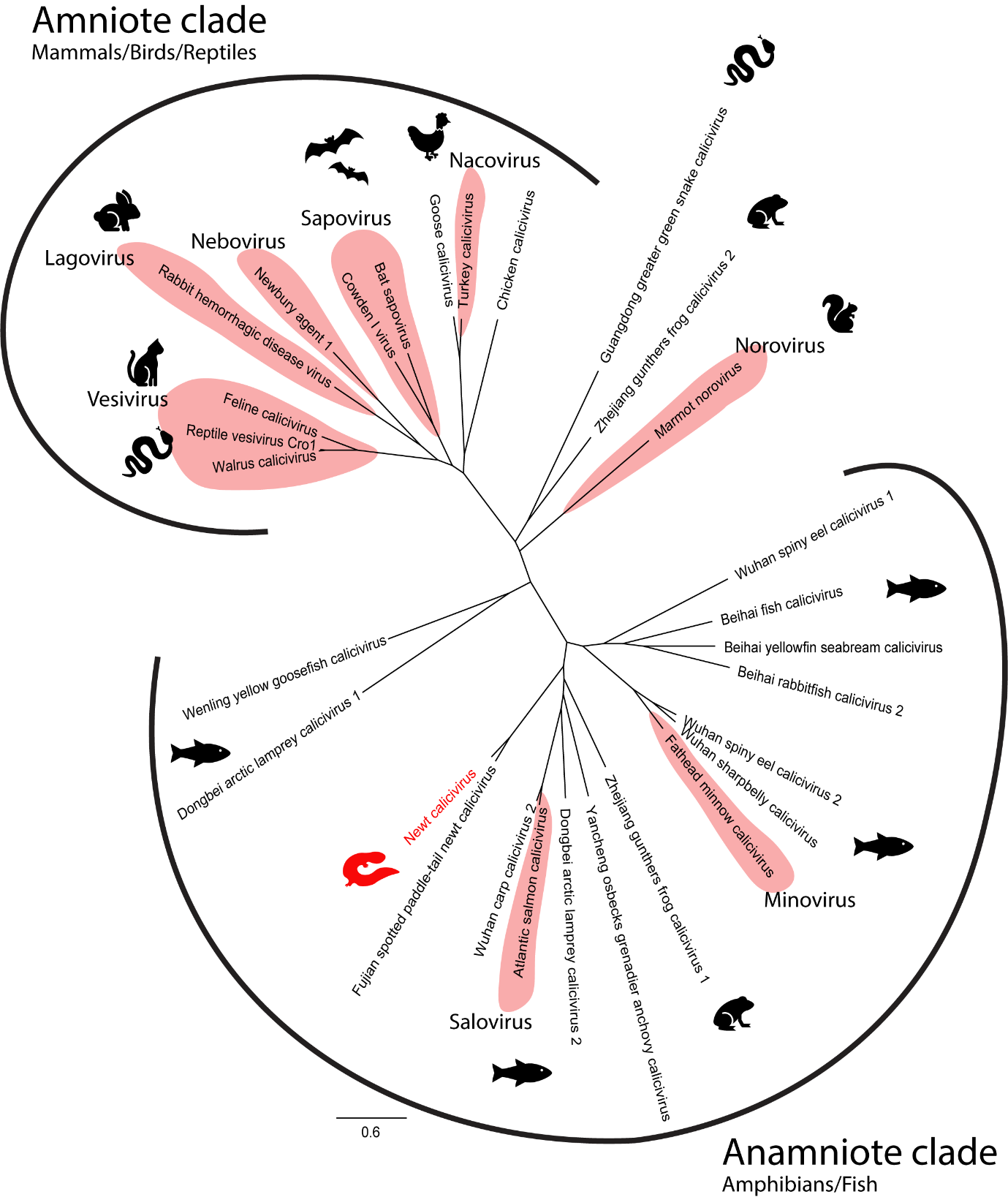


***Supplementary Figure 5 – Novel newt calicivirus.*** *Contigs were identified using a BLAST search of calicivirus proteins downloaded from NCBI protein database against herptile transcriptomes. The polyprotein (7390 nt) of newt calicivirus was* in silico *translated and aligned with reference orthomyxoviruses using MAFFT. A phylogenetic tree was constructed using RAxML with 500 bootstrap replicates. Newt calicivirus is coloured red and known classifications are shaded. The scale bar represents substitutions per site.*
